# Heronry distribution and site preference dynamics of tree-nesting colonial waterbirds in Tamil Nadu

**DOI:** 10.7717/peerj.12256

**Published:** 2021-10-07

**Authors:** Sadrack Jabaraj Dhanaraj Frank, Govindan Veeraswami Gopi, Bivash Pandav

**Affiliations:** Department of Endangered Species Management, Wildlife Institute of India, Dehradun, Uttarakhand, India

**Keywords:** Monsoon, Protected and non-protected areas, Wetlands, Anthropogenic disturbance, Heronry, Tree-nesting colonial waterbirds

## Abstract

Anthropogenic disturbance and climate change have significantly affected the distribution of wetlands globally and particularly in Asian countries. Various types of wetlands are harboured across all the biogeographic zones in India. These wetlands provide vital ecological services and are rich in biodiversity. However, anthropogenic pressures continue to be a threat to these wetlands by affecting the flora and fauna that depend on them. Tree-nesting colonial waterbirds are vulnerable to these pressures as their colonies are typically located in wetlands and associated areas. Disturbances to these areas have resulted in the loss or shifting of many heronries. The present study was conducted in the Indian state of Tamil Nadu during the period of 2017–2019 to document the existing and previously unknown heronries of the landscape. A total of 101 heronries were documented in 22 districts. The Little Cormorant was the most dominant species, occurring in 79% of the sites, with relative abundances of 24% and 26% during 2017–2018 and 2018–2019, respectively. A total of 23 tree species were utilized by the birds for nesting and *Vachellia nilotica* trees were used for nesting in about 25% of the heronries. 19% of the heronries were situated inside protected areas and 81% were located outside protected areas. Out of the 58 active nesting sites reported in 2005, 43 have been lost or are no longer active. Species distribution modelling with presence only data indicated that the sites with a high probability of occurrence were confined to the major waterbodies and rivers. Spatial correlation showed that the heronries were dispersed randomly across the landscape. The population dynamics within heronries and colonial nesting waterbirds’ response to various environmental factors must be monitored continuously to conserve these heronries.

## Introduction

Tree-nesting colonial waterbirds (hereafter “Colonial nesting waterbirds”) breed in single or mixed-species colonies (heronries). They play a significant role as ecological indicators of the health of a wetland ecosystem. Colonial nesting waterbirds help maintain the health of an ecosystem by acting as nutrient providers, bioindicators, and ecosystem engineers (Green & Elmberg, 2014). However, colonial nesting poses an important challenge for waterbird conservation since a large proportion of the population of a species may be concentrated in relatively small, isolated areas (Kushlan et al., 2002). Although colonial nesting waterbird species have different habitat preferences and diets, their nesting area requirements are similar (Hafner, 2000). Colonial breeding is an evolutionary puzzle and represents a trade-off between costs and benefits (Danchin & Wagner, 1997; Brown, 2016; Brzezinski et al., 2018). The costs include increased intraspecific competition for critical resources, disease transmission and attraction of predators; the direct benefits are increased foraging efficiency and resource localization (Alexander, 1974).

Wetlands across tropical and subtropical Asia are considered key centres as they provide significant ecosystem services and resources. Many local people are entirely dependent on these resources for their livelihoods (Ranga, 2006; Friend, 2007); this dependance highlights the importance of the conservation of wetlands (Sharma et al., 2019). However, degradation and loss of wetlands have raised serious concerns about waterbird populations worldwide (Beyersbergen, Niemuth & Norton, 2004; Bakker, 2005). Wetlands of diverse types are distributed across all the biogeographic zones in India (Islam & Rahmani, 2008). As per the Ramsar convention, both natural and manmade wetlands in India constitute the wetland ecosystem (Bassi et al., 2014). In India, wetlands, irrespective of location, size, ownership, biodiversity or ecosystem services, can be categorized under the Wetland (Conservation and Management) Rules 2017. River channels, paddy fields, human made waterbodies and wetlands falling within areas covered under Indian Forest Act 1927, Forest Conservation Act 1980, Wildlife Protection Act 1972 and Coastal Regularization Zone Notification, 2011 (Ministry of Environment Forest & Climate Change (MoEF&CC), 2020) are excluded. In addition to natural wetlands, extensive man-made wetlands also contribute to the rich faunal and floral diversity.

Wetland habitats are affected by anthropogenic activities (Hayal, Brook & Aramde, 2012) and the species dependent on them face tremendous pressure from factors such as unsustainable use of resources, pollution, anthropogenic pressure and disrupted flow regimes (Millennium Ecosystem Assessment, 2005). Some of these changes have impacted colonial nesting waterbirds, resulting in the loss of heronries (Roshnath & Sashikumar, 2019). Wetland birds, in particular colonial nesters, are known indicators of environmental change in their supporting ecosystem (Kushlan, 1993). Colonial nesting waterbirds breed in a select few locations. Even a small disturbance to those sites may have profound consequences on the waterbird populations. Continued management interventions are required to sustain these dynamic sites for long term conservation.

In the last century, many heronries across the Indian landscape have been lost (Subramanya, 1996). Developing conservation strategies for these ecological indicator species (Ogden et al., 2014), requires assessment of their current nesting and distribution patterns. Maintaining a viable population of colonial nesting waterbirds largely depends on regular site-specific wetland management interventions as these waterbirds exhibit strong site fidelity. Disturbance at nesting sites affect nesting behaviour which results in abandonment of the site (Carney & Sydeman, 1999; Roshnath & Sinu, 2017). Climate change causes major shifts in the features of waterbird habitats (Wormworth & Mallon, 2006) and, in conjunction with irregular monsoons, has altered the distribution of their nesting habitats (Urfi, 2011, Jabaraj & Gopi, 2020). Birds may physiologically respond to changes in temperature and precipitation caused by climate change (Steen & Powell, 2012; Pavón-Jordán et al., 2019). Thus, information related to nesting locations and breeding periods is vital in long term monitoring of waterbirds in relation to the current climate change (Urfi, 2011).

Tamil Nadu has some of the largest breeding habitats of colonial nesting waterbirds (Subramanya, 2005), with some nesting sites being centuries old (Shortt, 1865; Rhenius, 1907). The state features many water bodies in the plains that serve as irrigation tanks (Venkatachalam & Balooni, 2018). Prior to this study, Subramanya’s study on the heronries of Tamil Nadu (Subramanya, 2005) conducted more than 15 years prior, was the most recent systematic inventory of the state’s waterbird colonies. A state-wide survey of waterbird breeding colonies was conducted to highlight the ecological and conservation significance of several wetlands that are outside the scope of the Wetland Rules. The specific objectives of the present study were (i) to conduct a state-wide survey to document the existing heronries, (ii) to document the relative abundance of the colonial nesting waterbird species across the state, (iii) to predict possible nesting areas by using species distribution models (SDMs) as prioritising conservation and management actions require reliable information and (iv) to document the threats faced by the nesting habitats. The current study attempts to understand how the heronries are distributed across a large landscape. The results indicate how the colonial nesting waterbirds are faring in the state and provide a concrete baseline for future work.

## Materials & methods

### Study area

**Figure 1:**
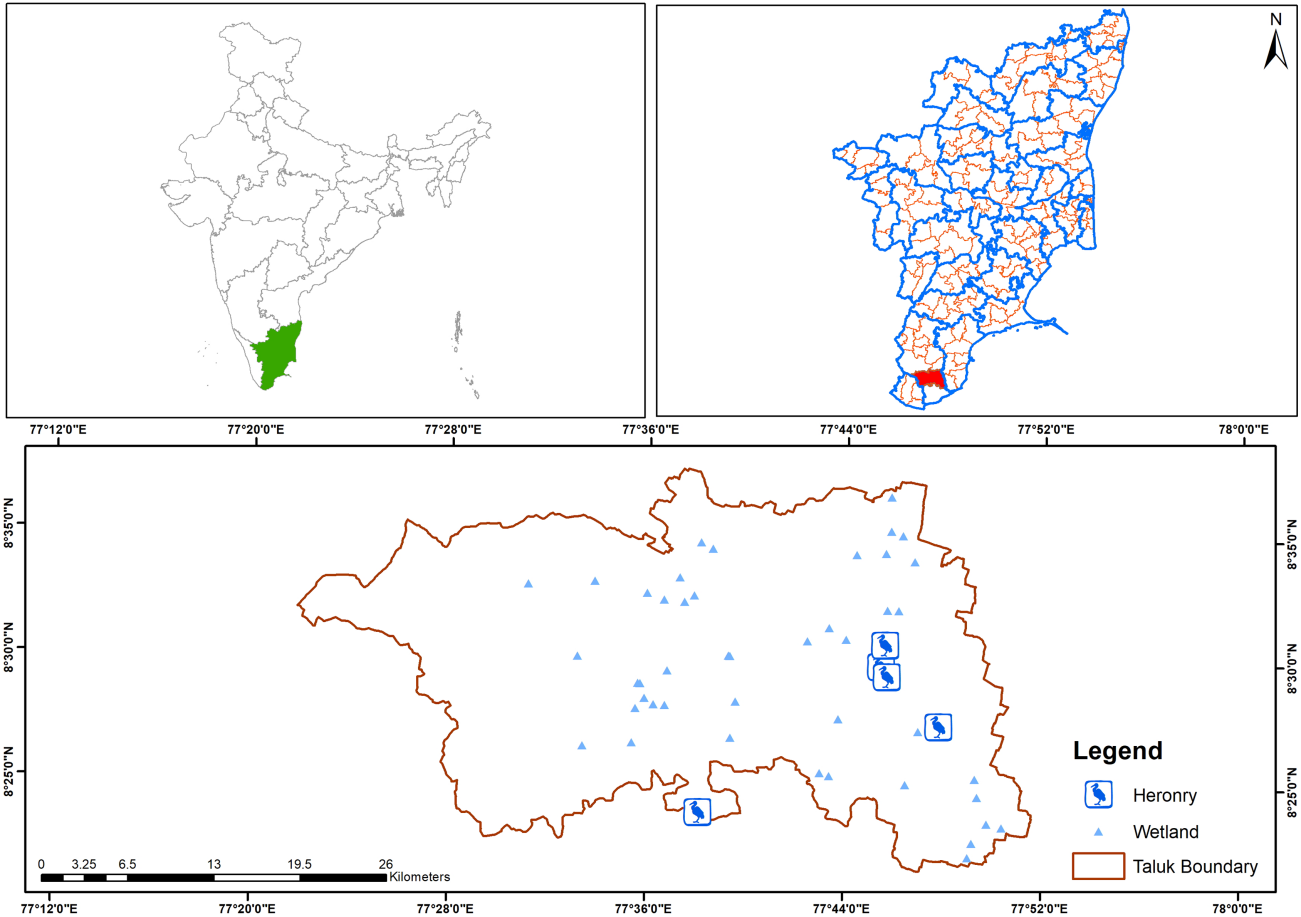
Map of the study area (Tamil Nadu).

The study was conducted in Tamil Nadu, which is broadly divided into three physiographic divisions: the north-western high-elevation regions, the western hilly area and the plains along the coast (Subramanya, 2005). The climate is semi-arid in the plains and humid to sub-humid in the hills. For administrative purposes, the state is divided into 32 districts and seven agro-climatic zones (Fig. 1). Whereas the rest of the country receives most of its rainfall during the south-west monsoon (SWM), *i.e*., from June to September, Tamil Nadu gets its rainfall during the north-east monsoon (NEM), *i.e*., from October to December. The state of Tamil Nadu lies in the rain shadow region of the Western Ghats due to which it receives limited rainfall during the SWM (June–September). The average annual rainfall of the state is about 921 mm and is largely dependent on the NEM to replenish its bodies of water (Subramanya, 2005; Singh et al., 2019). Both the livelihoods of the local people and the breeding cycles of the colonial nesting waterbird species are affected, if there is a monsoon failure (Jabaraj & Gopi, 2020).

The state has 17 river basins (Palanisami et al., 2011) and about 7% of the geographic area of Tamil Nadu is comprised of wetlands (rivers and reservoirs) (SAC, 2011). A total of 24,684 inland wetlands have been mapped at a scale of 1:50,000 in Tamil Nadu (SAC, 2011). The major wetland types in the state are rivers, lakes, reservoirs, ponds and tanks (SAC, 2011). Tanks are small and have mainly been constructed for storing water for household and irrigation purposes (Subramanya, 2005), whereas reservoirs include dams and lakes and are larger in size. The tanks in India are considered as the largest source of irrigation and they are highly concentrated in the southern states.

The tanks across the state, especially those near dams, store water during the lean season and supply water for agricultural purposes, mainly rice cultivation (Von Oppen & Rao, 1980). These tanks, along with the rivers, reservoirs and coastal wetlands, serve as excellent feeding, roosting and nesting grounds for colonial nesting waterbirds and as stopovers for migratory birds. This study was carried out during the period from 2017–2019. The two-study period was defined as the first year (October-2017 to September-2018) and the second year (October-2018 to September-2019). The survey was conducted predominantly at each district with further focus at the taluk (subdivision of a district) level. Initially, literature was studied to identify already existing heronries. A Survey of India map (1:50,000) was additionally used. Waterbodies were located using Google Earth and were surveyed for the presence of breeding colonies of colonial nesting waterbirds. A total of 123,486 km was travelled across the state during the study period. The information about the congregation of waterbirds was obtained from eBird (2017). The XXVII meeting of the Training, Research and Academic Council (TRAC) of the Wildlife Institute of India approved this work (21-02-2012). The Principal Chief Conservator of Forests and Chief Wildlife Warden approved a permit to enter protected areas for this research (Ref No. WL5(A)/19603, Permission No. 32/2017, Dated 07-07-2017).

### Waterbird survey

Waterbirds in the breeding colonies were identified and counted, and information pertaining to the GPS location of the heronries, number of species nesting, details of the location and nesting trees was documented in the prescribed format. When counting colonial nesting waterbirds, it is most important to consider the fluctuations in the number of adult birds during the day. Colonial nesting waterbirds spend a significant part of the time outside the nesting area, either for foraging or collecting nesting material. Hence, the count must be carried out at the same time of each day at a particular site. The standard counting protocol used for colonial nesting waterbirds, *i.e.*, total counts, was followed at each nesting site (Bibby et al., 2000). A vantage point was selected at each site, and the count was carried out in the morning before the birds had left the heronry (between 0500 and 0700 h) and repeated in the evening (between 1600 and 1800 h).

One of the major concerns was the detectability of the waterbird colonies. The survey involved a large geographical area, hence there was a high probability of missing breeding locations. To address this issue, local birdwatchers and non-governmental organizations (NGOs) assisted with locating known waterbird nesting colonies. One of the main objectives of the study was to locate previously unknown colonies and maximum effort and time was invested in reporting potential waterbird nesting colonies across the landscape. Two major challenges were the probability of missing a nesting colony if the area was inaccessible (*e.g.*, aquatic habitats) or that the timing of the survey was incorrect for the target species. This was overcome by using country boats to survey waterways and aquatic habitats that were not accessible by roads and conducting the survey during both the monsoons (SWM and NEM). Moreover, standard protocols pertaining to colonial waterbirds survey were followed (Steinkamp et al., 2003; Cavitt et al., 2014).

### Species distribution modelling

The suitability of sites for heronries was modelled using MaxEnt (Phillips, Anderson & Schapire, 2006). The global climate database WorldClim was used to acquire climate-related data (www.worldclim.org). Cumulative temporal weather data (1950–2000) was used to calculate the mean annual temperature, and a raster of resolution one km was extracted. The average precipitation (in millimetres) during the peak breeding season of the colonial nesting waterbirds, elevation (in metres), forest cover, land use/land cover and night-time light data (one-km resolution) were obtained (Table S1). The precipitation, temperature, forest cover and elevation were used as covariates as there was no pattern in the land use/land cover and night-time light data. To ensure better performance with the smaller data set, fivefold cross-validation was used, with the area under the curve (AUC), to assess the model. The regularization was kept at the default value of 1.0 to avoid over-fitting. The best-fitting model was chosen using the jack-knife variable selection procedure.

### Data analyses

The relative abundances of the colonial nesting waterbirds were calculated for the two survey periods. The relative abundance (R) of waterbirds was calculated using the formula

R = (Ni/Tn) ∗ 100

where,

Ni = total number of individuals of the ith species

Tn = total number of individuals of all species

The species accumulation curve was used to evaluate the relationship between the sampling effort and species detection over the survey period. Moran’s I index was used with a Euclidian distance estimator to check whether the sites were clustered, random or dispersed (Getis, 2008). All the statistical analyses were performed using the statistical program R (R Core Development Team, 2019).

## Results

**Figure 2:**
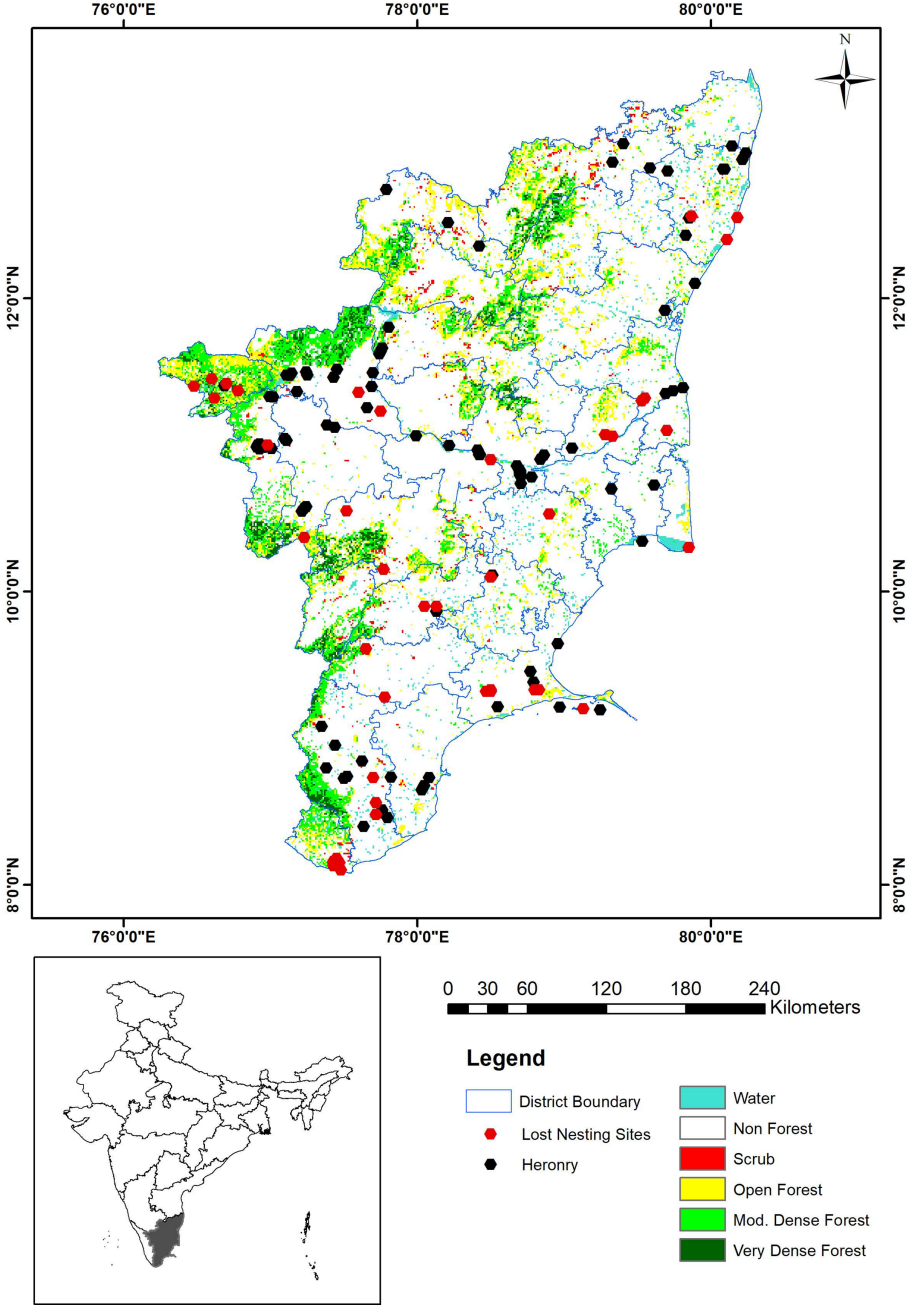
Map showing the active and lost heronries (2017–2019).

A total of 101 heronries were identified as active nesting sites in Tamil Nadu during the study period (Fig. 2). Among the 32 districts in the state, 22 had heronries. The maximum number of heronries were in Erode District (16 heronries), Tiruchirappalli District (14 heronries), Tirunelveli (12 heronries), Coimbatore (11 heronries) and Kancheepuram (nine heronries) (Table S2). There was one site in the union territory of Pondicherry. Out of the 58 active nesting sites reported in 2005 (Subramanya, 2005), 43 have been lost or are no longer active (Fig. 2).

**Figure 3:**
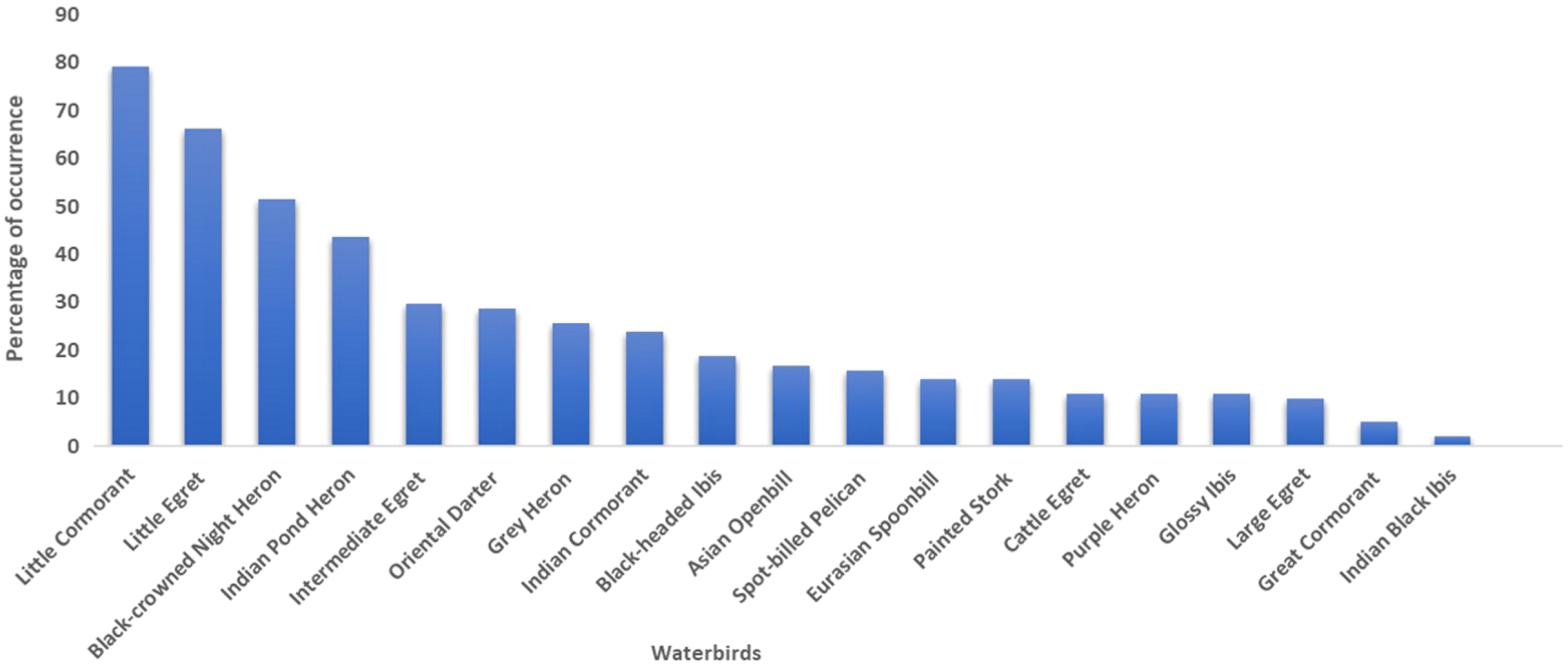
Nesting percentage of colonial nesting waterbirds among the total observed sites in Tamil Nadu (*n* = 101sites).

Among the 26 colonial nesting waterbird species of India (Subramanya, 1996) (Table S3), 19 (belonging to the families Pelecanidae, Ardeidae, Anhingidae, Ciconiidae, Threskiornithidae and Phalacrocoracidae) occur in Tamil Nadu (Table 1). Four of these 19 species are categorized as Near Threatened on the International Union for Conservation of Nature (IUCN) Red List: Oriental Darter, Spot-billed Pelican, Black-headed Ibis and Painted Stork. The Little Cormorant was found to be the most commonly occurring species, followed by the Little Egret, Black-crowned Night Heron and Indian Pond Heron. The least common species of waterbird in Tamil Nadu was the Indian Black Ibis, followed by the Great Cormorant and Large Egret (Fig. 3). The Black-crowned Night Heron was the dominant nesting species during the earlier study but has now been replaced by the Little Cormorant.

**Table 1:** List of Colonial nesting waterbirds breeding in the state of Tamil Nadu.

Common name	Scientific name	Status
Spot-billed Pelican	*Pelecanus philippensis*	NT
Little Egret	*Egretta garzetta*	LC
Intermediate Egret	*Ardea intermedia*	LC
Large Egret	*Ardea alba*	LC
Cattle Egret	*Bubulcus ibis*	LC
Grey Heron	*Ardea cinerea*	LC
Purple Heron	*Ardea purpurea*	LC
Indian Pond-Heron	*Ardeola grayii*	LC
Black-crowned Night-Heron	*Nycticorax nycticorax*	LC
Glossy Ibis	*Plegadis falcinellus*	LC
Black-headed Ibis	*Threskiornis melanocephalus*	NT
Indian Black Ibis	*Pseudibis papillosa*	LC
Eurasian Spoonbill	*Platalea leucorodia*	LC
Little Cormorant	*Microcarbo niger*	LC
Indian Cormorant	*Phalacrocorax fuscicollis*	LC
Great Cormorant	*Phalacrocorax carbo*	LC
Oriental Darter	*Anhinga melanogaster*	NT
Painted Stork	*Mycteria leucocephala*	NT
Asian openbill	*Anastomus oscitans*	LC

**Note:**

NT, Near Threatened; LC, Least concern.

**Figure 4:**
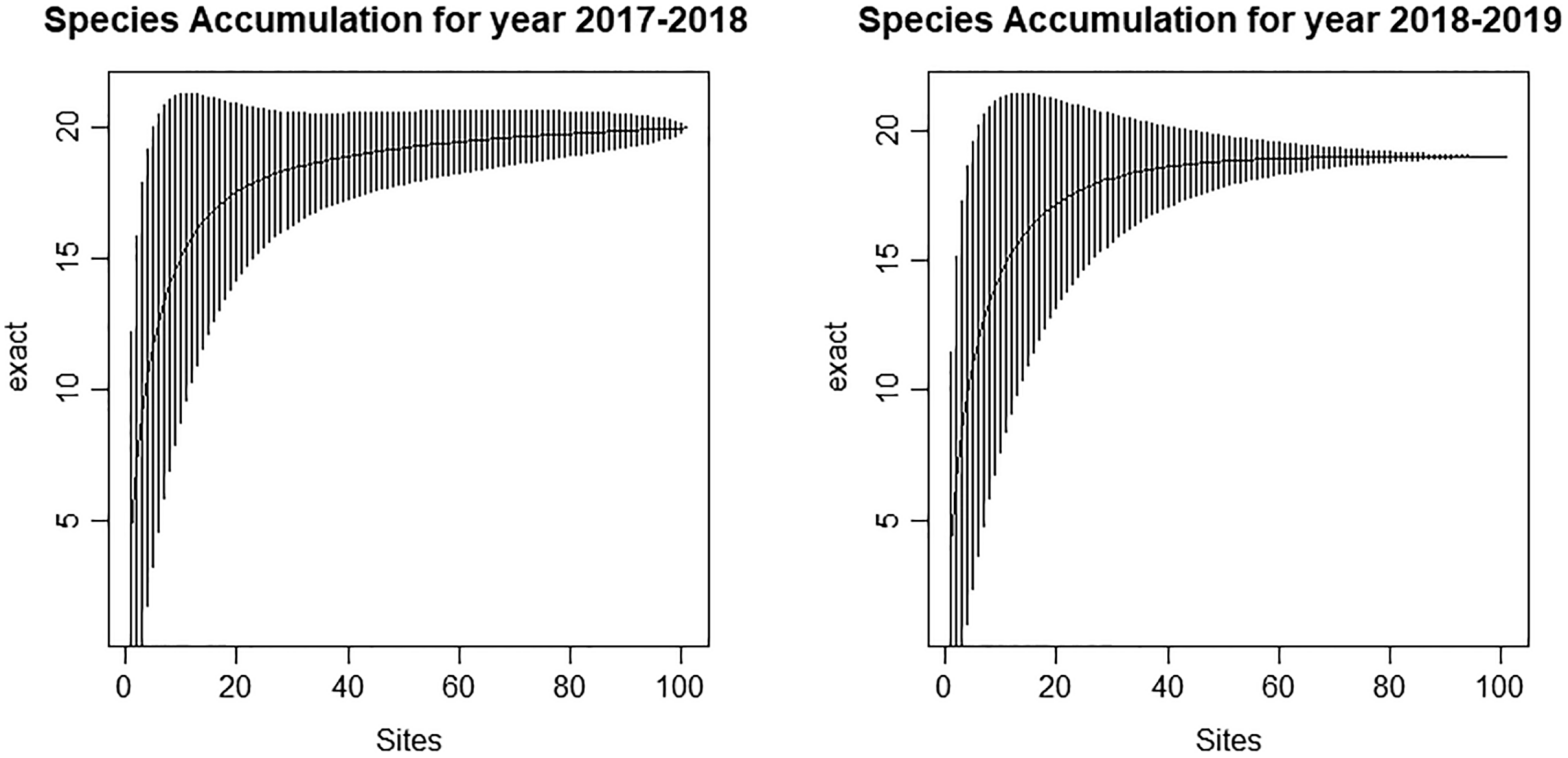
Species accumulation curve of heronry birds detected during 2017–2018 and 2018–2019 in the heronries of Tamil Nadu.

The relative abundances of the species were significantly different between the first survey year and the second survey year (Table 2). The species diversity of the community was compared randomly using species accumulation curve, and there was very little difference between the two survey periods (2017–2018, 2018–2019). The species accumulation curve over the 2-year period was adequate to detect the species in the study area (Fig. 4).

**Table 2:** Relative abundance of colonial nesting waterbirds in 2017–2018 and 2018–2019.

Species	rel_abun (%)	rel_abun (%)
Asian openbill	5.98	2.73
Eurasian Spoonbill	0.88	0.65
Little Cormorant	23.81	25.89
Indian Cormorant	8.35	7.97
Great Cormorant	0.39	0.85
Little Egret	5.17	4.60
Intermediate Egret	2.06	1.36
Large Egret	0.31	0.21
Cattle Egret	6.28	7.64
Purple Heron	0.19	0.16
Black-crowned Night-Heron	7.54	7.22
Indian Pond-Heron	3.46	3.81
Grey Heron	1.42	2.02
Indian Black Ibis	0.16	0.42
Glossy Ibis	12.47	16.76
Painted Stork	7.95	5.47
Spot-billed Pelican	7.10	6.16
Black headed Ibis	5.34	4.87
Oriental Darter	1.14	1.22

A total of 19 of the observed heronries are located within protected areas (bird sanctuaries, conservation reserves and national parks) and 82 heronries are outside protected areas (Table S2). Of the 81.19% non-protected heronry sites, 20.79% are in areas that are not legally protected, and are protected by people. A total of 23 tree species were utilized by the birds for nesting. Among the tree species, *Vachellia nilotica* was predominantly used by colonial waterbirds for nesting (Table 3). Apart from natural nesting trees and snags, artificial nesting structures (<1%) (Jabaraj & Gopi, 2021) were also used by the waterbirds. Most of the breeding coincided with the onset of the NEM (October–December). Some of the breeding coincided with the release of water in some of the major dams and reservoirs in the state during the SWM (Table S4).

**Figure 5:**
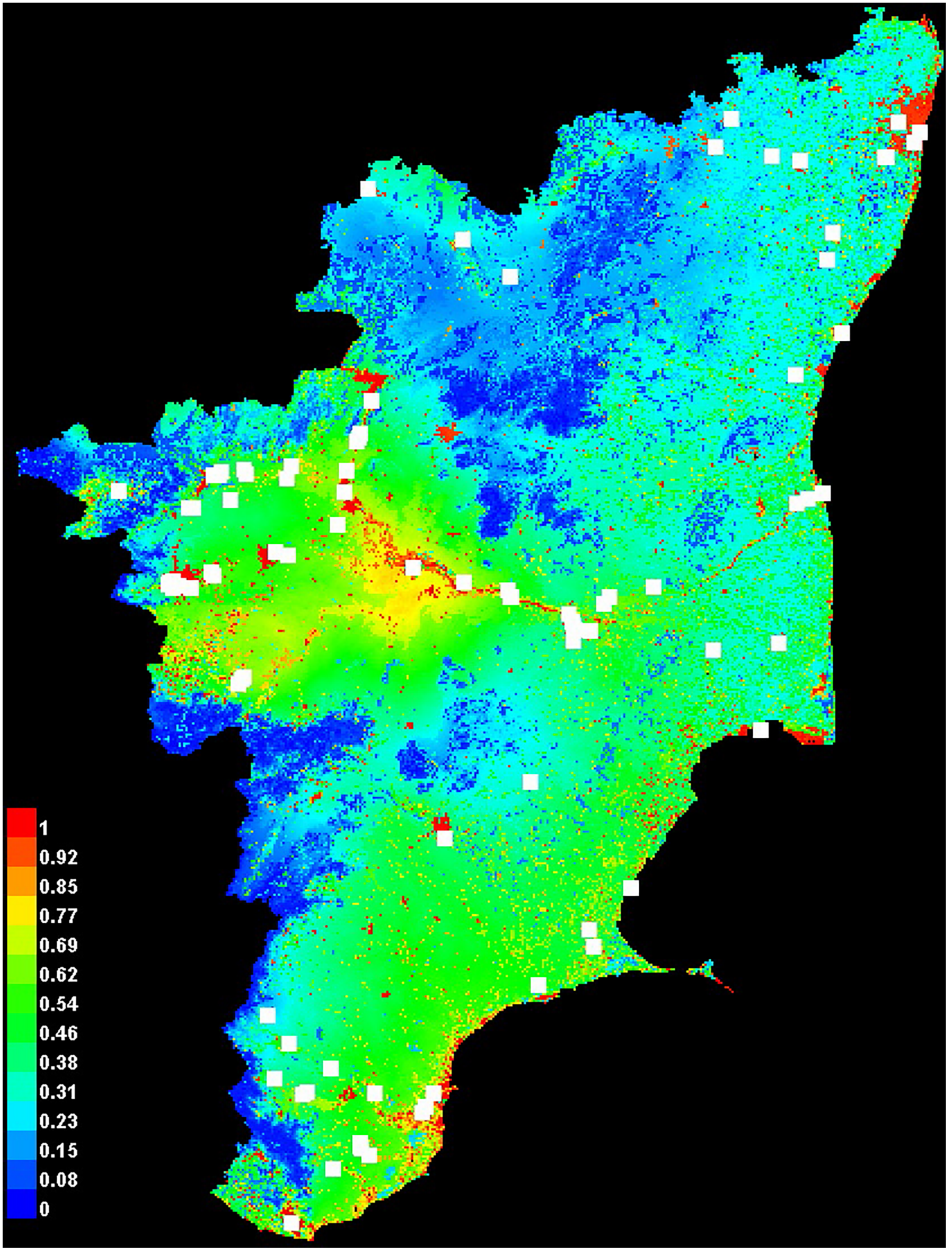
Protection status of the habitats (PA, Protected area; Non-PA, Non-Protected area and NLP, Non legally protected).

**Table 3:** Utilization of tree species for colonial nesting waterbirds among the total observed sites in Tamil Nadu.

Tree species used for nesting	Percentage of heronries (101 sites)
*Vachellia nilotica*	24.75
*Prosopis juliflora*	17.82
*Tamarindus indica*	12.87
*Ficus religiosa*	7.92
*Peltophorum pterocarpum*	4.95
*Avicennia marina*	3.96
*Azadirachta indica*	3.96
*Borassus flabellifer*	3.96
*Bambusa bambos*	2.97
*Barringtonia acutangula*	2.97
*Mangifera indica*	2.97
*Albizia saman*	1.98
*Madhuca longifolia*	1.98
*Eucalyptus globulus*	1.98
*Pithecellobium dulce*	1.98
*Pandanus odorifer*	<1
*Polyalthia longifolia*	<1

The nesting trees were predominantly found to be situated in wetlands (partially submerged in the water or around the wetlands), within campuses (mainly government offices, research institutions, factories and temples), amidst human habitations, along major highways (national highways/state highways), in mangrove vegetation, on islands and along the banks of large lakes and rivers (Table 4).

**Table 4:** Habitat details of the heronries during the period from 2017–2019.

Habitat	Inside protected area	Outside protected area	Total no. of heronry sites
Wetland	12	35	47
Within campuses	0	17	17
Human habitation	1	8	9
Religious area	0	3	3
Roadside (National highway/State highway)	0	12	12
Mangroves	3	1	4
Islands	2	0	2
Aviary	1	0	1
Rivers/lakes banks	0	6	6

The MaxEnt species distribution model that was developed predicted possible habitats for colonial nesting waterbirds in Tamil Nadu. All the known heronries were included to increase accuracy and the map with the probable areas is shown in Fig. 5. The heronries were analysed to determine if there was spatial correlation in the landscape. The results showed that the heronries were not spatially structured and seem to be randomly dispersed in space (Moran’s I = 0.049028, z score =1.901437, *p*-value = 0.057245).

## Discussion

### Distribution of heronries

The highest number of heronries were recorded in Tirunelveli, Ramanathapuram and Kancheepuram districts, with 12 heronries in each as per the previous study (Subramanya, 2005). As per Ganesh, Abhisheka & Prashanth (2014), Tirunelveli and Thoothukudi accounted for ten heronries. In the present study, Ramanathapuram district accounted for only six active heronries. Eight districts had only one heronry each and heronries were not recorded in ten districts of the state during the study period. Active nesting sites were recorded in Salem and Karur districts, which were devoid of heronries in earlier studies. More than 70% of heronries, that were active for the past 3–5 years, were reported for the first time in the present study. Three sites, Madras Crocodile Bank Trust, Dhamal and Samantham tank, that were not active during the earlier study were found to host colonial nesting waterbirds during the current study.

An earlier study, which also included erstwhile heronries, reported 97 heronries, but only 58 active nesting sites were recorded during that study (Subramanya, 2005). A total of 15 sites were common to the present study which included 101 sites and the earlier study which included 58 active sites. Continuous monitoring is required to understand the relationship between colonial nesting waterbirds and the environmental factors and habitat quality, particularly at the nesting areas (Ismail & Rahman, 2012). The biggest challenge in addressing the occupancy of waterbird species and their population dynamics is the lack of studies on landscape effects, especially in Tamil Nadu. For species that often change breeding sites, conservation and management interventions should be taken at a landscape level rather than at specific sites (Wyman, Wires & Cuthbert, 2014). As these birds are subjected to variety of threats, a landscape level approach in terms of monitoring and surveys should be regularly conducted during the peak breeding season across the region.

### Nesting pattern of colonial nesting waterbirds

The Little Cormorant was recorded in about 80 heronries, both in protected and non-protected areas. It was also found outside wetlands, especially along roadsides, as a single-nesting species or with two to four species. The Little Egret was found at 67 nesting sites, the Black-crowned Night Heron at 52 and the Indian Pond Heron at 44. The Oriental Darter nested at 29 sites, which is significantly higher than previously recorded. The Spot-billed Pelican nested at 16 sites and the Black-headed Ibis at 19 sites. The Indian Black Ibis was observed as the least common nesting species in Tamil Nadu (Table S5). The Great Cormorant was next, nesting in five heronries. Our low detection of species such as the Cattle Egret and Indian Black Ibis could be due to the fact that they normally prefer to nest away from mixed-species heronries in this landscape, though, the Cattle Egret is known to breed in monospecific and mixed colonies (Dwevedi, Kumar & Mylswamy, 2015). Species such as the Painted Stork, Spot-billed Pelican, Black-headed Ibis, Asian Openbill, Eurasian Spoonbill and Great Cormorant nested at well protected sites. Except for two sites, the Painted Stork nested predominantly in bird sanctuaries. Colonies of this species as well as the Spot-billed Pelican were found only at sites that enjoyed the utmost protection. In most of the heronries, the nesting of the Spot-billed Pelican began before the Painted Stork contrary to the previous study (Subramanya, 2005). Koonthankulam is known to host the Spot-billed Pelican; the oldest record goes back many centuries (Webb-Peploe, 1945). These reports indicate that the Spot-billed Pelican has nested in the southern states for more than a century and that these states have become a traditional home for this species (Subramanya & Manu, 1996; Subramanya, 1996, 2005). A total of 11 of the 17 nesting sites of the Asian Openbill were in the districts on the east coast, and six nesting sites were in the inland districts of Tamil Nadu.

The Great Cormorant has been reported to nest around reservoirs in the Nilgiris District, Koonthankulam Bird Sanctuary and Vedanthangal Bird Sanctuary (Subramanya, 2005), but nesting was not observed at either of these two bird sanctuaries in the present study. Active nesting sites were observed at three bird sanctuaries, Vaduvoor, Karaivetti, Vellode, and at one site each in the Nilgiris and Krishnagiri Districts. All the three ibis’ species (Black-headed Ibis, Indian Black Ibis and Glossy Ibis) breed in Tamil Nadu (Subramanya, 2005); the Black-headed Ibis is the most common of the three ibis’ species, followed by the Glossy Ibis. Interestingly, the Indian Black Ibis was never found nesting along with other species during the present study and was only observed breeding in Tirunelveli District. This species was first recorded nesting in Koonthankulam Bird Sanctuary (Wilkinson, 1961). From a conservation perspective, for species that nest away from wetlands, it is important to protect their traditional sites. For example, in the south-eastern coastal districts of Tamil Nadu, the Indian Black Ibis prefers the palmyra palm for building its nests. Both single-species and mixed-species heronries occur in Tamil Nadu. Two heronries had 16 species nesting—these were the oldest bird sanctuaries in Tamil Nadu (Vedanthangal and Koonthankulam). Ten heronries had 11 to 15 nesting species, and 18 sites had six to ten species. The majority (71 heronries) had one to five species, with around 16 of them being single species nesting colonies (Table S6). Sites that have large numbers of nesting species tend to be in bird sanctuaries or in areas where people actively protect the nests during the breeding season (*e.g*., Vagaikulam heronry) (Abhisheka et al., 2012).

### Nesting tree species

The habitat selection and population dynamics of colonial nesting waterbirds are influenced by various factors, among which nesting tree quality is one of the predominant criteria (Baxter & Fairweather, 1998; Roshnath & Sinu, 2017). *Vachellia nilotica* was found in most of the wetlands planted by the forest department in the early 1960s to meet the demand for fuelwood (Wilson, 1979). Most of the tanks in Tamil Nadu were planted with this species, which resulted in thick vegetation. *Vachellia nilotica* attracted waterbirds in large numbers and supported nesting activities. Before this species was planted, colony-nesting waterbirds built their nests amidst human habitations. They gradually shifted to the *Vachellia nilotica* plantations in the tanks (Subramanya, 2005). One example is Koonthankulam, where waterbirds initially preferred trees amidst human habitations and later shifted to the trees in the tanks.

Trees located along roadsides (national highways/state highways), near residential areas and in non-residential areas (inside campuses) have been recorded as excellent nesting habitats for colonial nesting waterbirds (Subramanya, 1996; Sashikumar & Jayarajan, 2007; Roshnath & Sinu, 2017). The present study corroborates this. *Tamarindus indica* and *Ficus religiosa* trees growing along roadsides, near reservoirs, along the banks of large rivers and in temple premises, provide excellent nesting habitats for the Little Cormorant, Little Egret, Indian Pond Heron and Black-crowned Night Heron. Tree species such as *Peltophorum pterocarpum* and *Delonix regia*, especially if located in campuses, provide conditions conducive to nesting. In general, many institutions and factories provide shelter to colonial nesting waterbirds. *Avicennia marina* was a mangrove species that was used by waterbirds extensively. Artificial nesting structures (Jabaraj & Gopi, 2021) have been constructed in recent years and are extensively utilized by colonial nesting waterbirds.

### Protected *vs*. non-protected heronries

One of the most effective measures employed in conservation is the establishment of legally protected areas (Dudley, 2008). The protected area network of Tamil Nadu includes bird sanctuaries, conservation reserves (areas owned by state governments adjacent to protected areas (PAs), for protecting the landscape, seascape and habitat of fauna and flora), reserve forests and islands. The species richness and diversity of waterbirds in the bird sanctuaries were higher in comparison with the rest of the protected area network (conservation reserves, reserve forests). Tamil Nadu has 15 bird sanctuaries and two conservation reserves. Colonial nesting waterbirds nest in nine of the bird sanctuaries and the two conservation reserves. During the study there was no nesting at four bird sanctuaries: Chitrangudi, Kanjirakulam, Sakkarakotai (Ramanathapuram District) and Karikili (Kancheepuram). A total of 82 heronries are situated outside protected areas and this is the biggest challenge for conservation of these heronries. Most areas with rich avifaunal biodiversity lie outside the legally protected areas. Urbanization is one factor that has changed the availability of nesting habitats (Lim & Sodhi, 2004; Urfi, 2010). Most of the nesting sites are dispersed within wetlands, along roadsides or in institutions and factories with no protection. Some of the sites located outside protected area networks enjoy protection through the efforts of local people (*e.g*., Vagaikulam heronry), the authorities of the factories (*e.g*., TVS motors), educational institutions and religious groups (Jabaraj & Gopi, 2021). Around 21 heronries fall in the non-legally protected area category (protected by people). There is minimal disturbance to the nesting colonial waterbirds, especially during the breeding season, and they are protected from hunting. In both years of the survey, the species abundance and richness were similar in the protected areas and the non-protected areas unless there was a failure of the monsoon and a dearth of water in a wetland (Jabaraj & Gopi, 2020). Waterbirds nesting inside campuses and religious premises are safeguarded from external disturbance; one good example is the Melmaruvathur heronry, where the colonial nesting waterbirds are given the utmost protection by the temple administration (Jabaraj & Gopi, 2021). Partnership between the local people and concerned authorities is critical in protecting the existing heronries located outside protected areas.

### MaxEnt: species distribution modelling

Breeding behaviour shaped by the climatic and anthropogenic changes in the landscape (Huntley et al., 2006) can be predicted using SDMs (Peterson, 2001). The MaxEnt species distribution model developed, predicted breeding habitats for colonial nesting waterbirds in Tamil Nadu. Presence-only data was used for the model; the predicted sites mostly clustered near waterbodies, along rivers and reservoirs and in areas where dense vegetation is close to waterbodies. The site-level variables required to understand the nesting ecology of waterbirds have still not been studied in depth (Kreakie, Fan & Keitt, 2012); parameters that could influence nesting include water depth, disturbance level, vegetation, distance from roads and settlements and water quality etc. (Maleki et al., 2016; Sheehan et al., 2017). Other landscape complexities that may also influence the nesting sites of these birds need to be identified and considered in future models.

The predicted hotspots were strongly corroborated (AUC: 0.75) by the field survey data. The wetlands in the high-probability areas were not strictly nesting sites but were used either as feeding or roosting sites, which can later be converted to nesting sites. Gurusami (1994) also observed this type of site behaviour. Environmental characteristics can be used to model the distribution of wildlife, and such modelling can help develop effective management plans to prioritize areas that may suffer major changes in a changing landscape (May, Page & Fleming, 2016). Due to the dynamic nature of the wetlands and our poor understanding of the impacts of climate change, it is hard to foretell the response of wetland species, especially waterbirds, to the ongoing landscape modifications and climate change. Moreover, climate change, which directly or indirectly influences the monsoon pattern, has a direct impact on the breeding colonies in a particular year (season), as is very evident from the present study. The breeding and the population are affected if the monsoon fails (Jabaraj & Gopi, 2020). Some of the sites located in human-dominated landscapes are completely at the mercy of the local administration or the people; in some cases, the wetlands are used for developmental activities (Panigrahy et al., 2012). To create better predictive models for larger landscapes further site-level studies must be carried out to understand the environmental variables that might influence the nesting behaviour of these birds.

### Threats

Habitat destruction (Fig. S1) and hunting (Fig. S2) remain the biggest threats to colonial nesting waterbirds. The novel techniques used by hunters targeting agricultural fields have become a major concern in recent years. Disturbance in the form of fishing (Fig. S3), collection of firewood and livestock grazing within the wetland affects the waterbirds nesting during breeding season. About 50% of the heronries recorded in the state occur outside wetlands among densely populated human habitation and along roadsides. Major threats to waterbird breeding include the felling of nesting trees during road widening, local residents reacting to the nuisance created by waterbirds, the collection of eggs from the nests and hunting. Species like Egrets, Herons and Cormorants are hunted across the state. Further encroachments within the wetlands and extensive guano deposition causing nesting tree death (Fig. S4) are other threats that must also be evaluated. The attitude of the people towards heronries also remains an important determining factor for the persistence of these species in this landscape. People occasionally drive away the birds before they start nesting by bursting crackers and other methods as they find guano excretion, noise, falling of the eggs and dead chicks to be a nuisance. Along with the above threats, climate change remains an important factor influencing these colonial nesting waterbirds (Cavitt et al., 2014).

## Conservation implications & conclusion

Tamil Nadu is an agrarian state (Lerche, 2014); water is extensively used for agriculture. Conducive habitat conditions can change within a short period of time, particularly in wetlands, which are prone to various disturbances. Tamil Nadu Forest Department implemented plantations on the foreshore of waterbodies that has resulted in the creation of many bird sanctuaries in the state (Bhaskar & Vencatesan, 2015). In many bird sanctuaries, the sharing of land ownership by various departments, such as the Forest Department and Public Works Department, poses a challenge during the peak breeding season. Thus, necessitating an integrated approach among all the stakeholders, if conservation outcomes are to be achieved.

A wetland is managed solely for conservation of biodiversity by establishing conservation reserves/bird sanctuaries or for other multiple purposes, dependent upon the people’s requirements (Sundar et al., 2015). The management issues pertaining to unprotected wetlands with rich biodiversity are poorly understood (Chester & Robson, 2013). In heronries that are situated away from waterbodies amidst human habitations, there are challenges in maintaining the nesting activity as these sites are continuously exposed to anthropogenic pressure. Steps must be taken to safeguard heronries along major roads against urbanization and rapid road expansion projects (Table S7) (Sashikumar et al., 2015). Such development activities would directly impact the nesting colonies, which are mostly used by species such as the Little Cormorant, Indian Pond Heron, Black-crowned Night Heron, Little Egret and Intermediate Egret, and affect the population dynamics of these species. Educating people about the ecological importance of waterbirds will help create concern for the birds nesting amidst human habitations (Jabaraj, Pandian & Gopi, 2021).

In this study, the “landscape” essentially covers the entire state of Tamil Nadu. The state-wide assessment of heronries was long overdue. The variables that predict state-wide distributions are quite important and need to be articulated to both policy makers and those that implement policies in the field. Wetland management, unlike conventional PA management, involves multiple stakeholders like the district administration, public works department, forest department (minimally), the local communities and their elected representatives. Prioritizing wetlands based on their importance as nest sites would be the first step in incorporating nest protection into wetlands management and working plans. The current study has updated the information on heronries across the state of Tamil Nadu. Some of the nesting sites might have been missed during the survey, but a systematic approach was adopted. Continuous monitoring is required to understand the trends in colonial nesting waterbird breeding across the landscape and address long-term questions such as the impacts of climate change and land-use alteration on the waterbird communities in the state of Tamil Nadu.

## Supplemental Information

10.7717/peerj.12256/supp-1 Supplemental Information 1 Environmental and ecological covariates used in heronry distribution modeling

10.7717/peerj.12256/supp-2 Supplemental Information 2 Heronry details with district details and protection status

10.7717/peerj.12256/supp-3 Supplemental Information 3 26 colonial nesting waterbird species breeding in India (Subramanya, 1996)

10.7717/peerj.12256/supp-4 Supplemental Information 4 Month wise breeding details of colonial nesting waterbirds at heronries in Tamil Nadu

10.7717/peerj.12256/supp-5 Supplemental Information 5 Percentage of occurrence

10.7717/peerj.12256/supp-6 Supplemental Information 6 Heronries with breeding colonial nesting waterbird and species richness

10.7717/peerj.12256/supp-7 Supplemental Information 7 Details regarding the Sites along the roadsides

10.7717/peerj.12256/supp-8 Supplemental Information 8 Relative abundance raw data

10.7717/peerj.12256/supp-9 Supplemental Information 9 Habitat destruction (felling of trees)

10.7717/peerj.12256/supp-10 Supplemental Information 10 Hunting

10.7717/peerj.12256/supp-11 Supplemental Information 11 Fishing

10.7717/peerj.12256/supp-12 Supplemental Information 12 Dying of trees (guano deposition)

10.7717/peerj.12256/supp-13 Supplemental Information 13 Abstract in Tamil Abstract of the article in Tamil (unreviewed).

## Additional Information and Declarations

### Field Study Permissions

The following information was supplied relating to field study approvals (*i.e*., approving body and any reference numbers): The Principal Chief Conservator of Forests and Chief Wildlife Warden approved a permit to enter into protected areas to carry out research (Permission No. 32/2017).

### Data Availability

The following information was supplied regarding data availability: The data are available in the Supplemental Files.

## References

[ref-1] Abhisheka K, Seshadri KS, Prashanth MB, Ganesh T (2012). The Agasthyamalai landscape: land of mountains, wetlands and biodiversity. Sanctuary Asia.

[ref-2] Alexander RD (1974). The evolution of social behaviour. Annual Review of Ecology and Systematics.

[ref-3] Bakker KK (2005). South Dakota all bird conservation plan. Wildlife Division Report 2005–09.

[ref-4] Bassi N, Kumar MD, Sharma A, Pardha-Saradhi P (2014). Status of wetlands in India: a review of extent, ecosystem benefits, threats and management strategies. Journal of Hydrology: Regional Studies.

[ref-5] Baxter GS, Fairweather PG (1998). Does available foraging area location or colony character control the size of multispecies egret colonies?. Wildlife Research.

[ref-6] Beyersbergen GW, Niemuth ND, Norton MR (2004). Northern Prairie and Parkland waterbird conservation plan. A plan associated with the Waterbird Conservation for the Americas Initiative.

[ref-7] Bhaskar A, Vencatesan J (2015). Challenges of managing water bodies as bird sanctuaries in Tamil Nadu. Current Science.

[ref-8] Bibby CJ, Burgess ND, Hill DA, Mustoe S (2000). Bird census techniques.

[ref-9] Brown CR (2016). The ecology and evolution of colony-size variation. Behavioral Ecology and Sociobiology.

[ref-10] Brzezinski M, Chibowski P, Gornia J, Górecki G, Zalewski A (2018). Spatio-temporal variation in nesting success of colonial waterbirds under the impact of a non-native invasive predator. Oecologia.

[ref-11] Carney KM, Sydeman WJ (1999). A review of human disturbance effects on nesting colonial waterbirds. Waterbirds.

[ref-12] Cavitt JF, Jones SL, Wilson NM, Dieni JS, Zimmerman TS, Doster RH, Howe WH (2014). Atlas of breeding colonial waterbirds in the interior western United States.

[ref-13] Chester ET, Robson BJ (2013). Anthropogenic refuges for freshwater biodiversity: their ecological characteristics and management. Biological Conservation.

[ref-14] Danchin E, Wagner RH (1997). The evolution of coloniality: the emergence of new perspectives. Trends in Ecology & Evolution.

[ref-15] Dudley N (2008). Guidelines for applying protected area management categories.

[ref-16] Dwevedi R, Kumar A, Mylswamy M (2015). A monospecific colony of Cattle Egret *Bubulcus ibis* in agricultural landscape of central Uttar Pradesh. India Indian BIRDS.

[ref-17] eBird (2017). eBird: An online database of bird distribution and abundance. eBird, Cornell Lab of Ornithology, Ithaca, New York. https://ebird.org/region/IN-TN.

[ref-19] Friend R (2007). Securing sustainable livelihoods through wise use of wetland resources: reflections on the experience of the Mekong Wetlands biodiversity conservation and sustainable use programme, (MWBP).

[ref-20] Ganesh T, Abhisheka K, Prashanth B, Gopi GV, Hussain SA (2014). Conservation of heronries in the districts of Tirunelveli and Thoothukudi, Southern Tamil Nadu. Waterbirds of India, ENVIS Bulletin: Wildlife & Protected Areas.

[ref-21] Getis A (2008). A history of the concept of spatial autocorrelation: a geographer’s perspective. Geographical Analysis.

[ref-22] Green AJ, Elmberg J (2014). Ecosystem services provided by waterbirds. Biological Reviews.

[ref-23] Gurusami V (1994). Wetland birds: year-round breeding in the Simpson Estate, Sembiam. Blackbuck.

[ref-24] Hafner H, Kushlan AJ, Hafner H (2000). Heron nest site conservation. Heron Conservation.

[ref-18] Hayal D, Brook L, Aramde F (2012). Aspects of climate change and its associated impacts on wetland ecosystem functions-A review. Journal of American Science.

[ref-25] Huntley B, Collingham YC, Green RE, Hilton GM, Rahbek C, Willis SG (2006). Potential impacts of climatic change upon geographical distributions of birds. Ibis.

[ref-26] Islam MZ, Rahmani AR (2008). Potential and existing Ramsar sites in India.

[ref-27] Ismail A, Rahman F (2012). Population dynamics of colonial waterbirds in upper Bisa, Putrajaya Wetlands, Malaysia. Acta Biologica Malaysiana.

[ref-28] Jabaraj DFS, Gopi GV (2020). At the behest of rainfall: a case of heronry formation failure in Tamil Nadu. Bird-o-soar #43. Zoo’s Print.

[ref-29] Jabaraj DFS, Gopi GV (2021). A case of successful initiative for heronry conservation in a privately managed wetland. Indian BIRDS.

[ref-30] Jabaraj DFS, Pandian M, Gopi GV (2021). Nest-site characteristics of an urban heronry at Ranipet Police Station, Tamil Nadu. Indian BIRDS.

[ref-31] Kreakie BJ, Fan Y, Keitt TH (2012). Enhanced migratory waterfowl distribution modeling by inclusion of depth to water table data. PLOS ONE.

[ref-72] Kushlan JA (1993). Colonial waterbirds as bioindicators of environmental change. Colonial waterbirds.

[ref-32] Kushlan JA, Steinkamp MJ, Parsons KC, Capp J, Cruz MA, Coulter M, Davidson I, Dickson L, Edelson N, Elliot R, Erwin RM (2002). Waterbird conservation for the Americas: the North American waterbird conservation plan, Version 1.

[ref-33] Lerche J (2014). Regional patterns of agrarian accumulation in India 1. Indian Capitalism in Development.

[ref-34] Lim HC, Sodhi NS (2004). Responses of avian guilds to urbanization in a tropical city. Landscape and Urban Plan.

[ref-35] Maleki S, Soffianian AR, Koupaei SS, Saatchi S, Pourmanafi S, Sheikholeslam F (2016). Habitat mapping as a tool for water birds conservation planning in an arid zone wetland: the case study Hamun wetland. Ecological Engineering.

[ref-36] May TM, Page MJ, Fleming PA (2016). Predicting survivors: animal temperament and translocation. Behavioral Ecology.

[ref-37] Millennium Ecosystem Assessment (2005). Ecosystems and human well-being: wetlands and water synthesis.

[ref-38] Ministry of Environment Forest &Climate Change (MoEF&CC) (2020). Guidelines for implementing wetlands (conservation and management) rules, 2017.

[ref-39] Ogden JC, Baldwin JD, Bass OL, Browder JA, Cook MI, Frederick PC, Lorenz JJ (2014). Waterbirds as indicators of ecosystem health in the coastal marine habitats of southern Florida: 1. selection and justification for a suite of indicator species. Ecological Indicators.

[ref-40] Palanisami K, Ranganathan CR, Vidhyavathi A, Rajkumar M, Ajjan N (2011). Performance of agriculture in river basins of Tamil Nadu in the last three decades-a total factor productivity approach. A Project Sponsored by Planning Commission, Government of India.

[ref-41] Panigrahy S, Murthy TVR, Patel JG, Singh TS (2012). Wetlands of India: inventory and assessment at 1:50,000 scale using geospatial techniques. Current Science.

[ref-42] Pavón-Jordán D, Clausen P, Dagys M, Devos K, Encarnaçao V, Fox AD, Frost T, Gaudard C, Hornman M, Keller V, Langendoen T (2019). Habitat-and species-mediated short-and long-term distributional changes in waterbird abundance linked to variation in European winter weather. Diversity and Distributions.

[ref-43] Peterson AT (2001). Predicting species geographic distributions based on ecological niche modeling. The Condor.

[ref-44] Phillips SJ, Anderson RP, Schapire RE (2006). Maximum entropy modeling of species geographic distributions. Ecological Modelling.

[ref-45] R Core Development Team (2019). A language and environment for statistical 309 computing.

[ref-46] Ranga MR (2006). Transformation of coastal wetland agriculture and livelihoods in Kerala, India.

[ref-47] Rhenius CE (1907). Pelicans breeding in India. Journal of Bombay Natural History Society.

[ref-48] Roshnath R, Sashikumar C (2019). Conservation challenges of the heronries in Kerala. Journal of the Bombay Natural History Society.

[ref-49] Roshnath R, Sinu PA (2017). Nesting tree characteristics of heronry birds of urban ecosystems in peninsular India: implications for habitat management. Current Zoology.

[ref-50] Space Application Centre (SAC) (2011). National Wetland Atlas.

[ref-51] Sashikumar C, Jayarajan O (2007). Census of the heronries of North Kerala. Malabar Trogon.

[ref-52] Sashikumar C, Bimalnath KG, Hari M, Harikumar M, Praveen ES, Raju S, Roshnath R, Sathyan M, Sreekumar B, Sivakumar AK, Vishnudas CK (2015). Heronries of Kerala. Malabar Trogon.

[ref-53] Sharma P, Baruah J, Deka D, Kaushik P (2019). Harnessing wetlands for sustainable livelihood.

[ref-54] Sheehan KL, Esswein ST, Dorr BS, Yarrow GK, Johnson RJ (2017). Using species distribution models to define nesting habitat of the eastern meta-population of double-crested cormorants. Ecology and Evolution.

[ref-55] Shortt J (1865). Account of a heronry, and breeding-place of other water-birds, in southern India. Journal of the Proceedings of Linnean Society of London, Zoology.

[ref-56] Singh AK, Tripathi JN, Kotlia BS, Singh KK, Kumar A (2019). Monitoring groundwater fluctuations over India during Indian summer monsoon (ISM) and Northeast monsoon using GRACE satellite: impact on agriculture. Quaternary International.

[ref-57] Steen V, Powell AN (2012). Potential effects of climate change on the distribution of waterbirds in the Prairie Pothole Region. USA Waterbirds.

[ref-58] Steinkamp M, Peterjohn B, Byrd V, Carter H, Lowe R (2003). Breeding season survey techniques for seabirds and colonial waterbirds throughout North America.

[ref-59] Subramanya S (1996). Distribution, status and conservation of Indian heronries. Journal of Bombay Natural History Society.

[ref-60] Subramanya S, Manu K (1996). Saving the spot-billed Pelican: a successful experiment. Hornbill.

[ref-61] Subramanya S (2005). Heronries of Tamil Nadu. Indian Birds.

[ref-62] Sundar KG, Chauhan AS, Kittur S, Babu S (2015). Wetland loss and waterbird use of wetlands in Palwal district, Haryana, India: the role of agriculture, urbanization and conversion to fish ponds. Wetlands.

[ref-63] Urfi AJ (2010). Using heronry birds to monitor urbanization impacts: a case study of painted stork Mycteria leucocephala nesting in the Delhi zoo, India. AMBIO.

[ref-64] Urfi A (2011). Climate change and its impacts on Indian birds: monsoon phenology and monitoring heronry birds. Current Science.

[ref-65] Venkatachalam L, Balooni K (2018). Water transfer from irrigation tanks for urban use: can payment for ecosystem services produce efficient outcomes?. International Journal of Water Resources Development.

[ref-66] Von Oppen M, Rao KVS (1980). Tank irrigation in semi-arid tropical India. Part 1: historical development and spatial distribution.

[ref-67] Webb-Peploe CG (1945). Notes on a few birds from south of the Tinnevelly district. Journal of Bombay Natural History Society.

[ref-68] Wilkinson ME (1961). Pelicanry at Kundakulam, Tirunelveli district. Journal of Bombay Natural History Society.

[ref-69] Wilson J (1979). Social forestry in Tamil Nadu. Indian Forester.

[ref-70] Wormworth J, Mallon K (2006). Bird species and climate change: the global status report. Version 1.1. Report to World Wildlife Fund.

[ref-71] Wyman KE, Wires LR, Cuthbert FJ (2014). Colonial waterbird site occupancy dynamics reflect variation in colony site environments in the US Great Lakes. Journal of Great Lakes Research.

