# Supplementary material for: Heronry distribution and site preference dynamics of tree-nesting colonial waterbirds in Tamil Nadu
Source: PeerJ. 2021 Oct 7;9:e12256. doi: 10.7717/peerj.12256 (PMC8502450; doi:10.7717/peerj.12256)
Supplement: Supplemental Information 1 [file peerj-09-12256-s001.docx]

Table S1: Details of all environmental and ecological covariates used in heronry distribution modeling

| Variables | Data source | Units/Range |
| --- | --- | --- |
| Annual mean Temperature | WorldClim (<https://worldclim.org/data/bioclim.html>) | °C |
| Annual Precipitation |  | mm |
| Nightlight | Defence Metrological Satellite Programme-Operational Line Scan System | 0 to 63 |
| Elevation | ASTER DEM (<http://www.ngdc.noaa.gov/mgg/topo/globe.html>) | m |
| Forest cover | Forest cover data 2017, Forest Survey of India | m |
| Land use/ land cover | Bhuvan LULC data 2018  (https://bhuvan-app3.nrsc.gov.in/data/download/) | m |
