# Supplementary material for: Heronry distribution and site preference dynamics of tree-nesting colonial waterbirds in Tamil Nadu
Source: PeerJ. 2021 Oct 7;9:e12256. doi: 10.7717/peerj.12256 (PMC8502450; doi:10.7717/peerj.12256)
Supplement: Supplemental Information 2 [file peerj-09-12256-s002.docx]

**Heronries at district level and their protection status**

| S.No | Name of the Heronry | Status | District |
| --- | --- | --- | --- |
| 1 | Vedanthangal | PA | Kancheepuram |
| 2 | Melmaruvathur | Non-PA (NLP) | Kancheepuram |
| 3 | National Institute of Ocean Technology (NIOT) | Non-PA (NLP) | Kancheepuram |
| 4 | Madras Crocodile Bank Trust | Non-PA (NLP) | Kancheepuram |
| 5 | Indian Institute of Technology Madras | Non-PA (NLP) | Chennai |
| 6 | Otteri | PA | Kancheepuram |
| 7 | Ponneri | Non-PA | Kancheepuram |
| 8 | Sri Ramachandra Medical College | Non-PA (NLP) | Kancheepuram |
| 9 | Kaliveli | Non-PA | Villupuram |
| 10 | Kanadamangalam | Non-PA | Villupuram |
| 11 | Thengaithittu | Non-PA | Pondichery |
| 12 | Agaranallur (Banks of Kollidam River stretch) | Non-PA | Cuddalore |
| 13 | Varagur (Banks of Kollidam River stretch) | Non-PA | Cuddalore |
| 14 | Kodiyampalayam | Non-PA | Nagapattinam |
| 15 | Vaduvoor | PA | Tiruvarur |
| 16 | Udayamarthandapuram | PA | Tiruvarur |
| 17 | Tiruvarur University & surrounding | Non-PA (NLP) | Tiruvarur |
| 18 | Periyakanmoi | PA | Ramanathapuram |
| 19 | Therthangal | PA | Ramanathapuram |
| 20 | Muthupet | PA | Tiruvarur |
| 21 | Melselvanur-Keelaselvanur | PA | Ramanathapuram |
| 22 | Karankadu | PA | Ramanathapuram |
| 23 | Vaalai Island | PA | Ramanathapuram |
| 24 | Shingle Island | PA | Ramanathapuram |
| 25 | Sayarpuram | Non-PA | Thoothukudi |
| 26 | korampalam | Non-PA | Thoothukudi |
| 27 | Arumugamangalam | Non-PA | Thoothukudi |
| 28 | Koonthankulam | PA | Tirunelveli |
| 29 | Kadankulam | PA | Tirunelveli |
| 30 | Tirupadaimaruthur | PA | Tirunelveli |
| 31 | Vagaikulam | Non-PA (NLP) | Tirunelveli |
| 32 | Velankulam | Non-PA | Tirunelveli |
| 33 | Kariyandi | Non-PA | Tirunelveli |
| 34 | Aramaneri | Non-PA | Tirunelveli |
| 35 | Kandigaiperi | Non-PA | Tirunelveli |
| 36 | Manur | Non-PA | Tirunelveli |
| 37 | Arunthapatti | Non-PA | Tirunelveli |
| 38 | Mukkadal | Non-PA | Tirunelveli |
| 39 | Kadayanallur | Non-PA | Tirunelveli |
| 40 | Suchindram | PA | Kanyakumari |
| 41 | Sulur | Non-PA | Coimbatore |
| 42 | Vellalore | Non-PA | Coimbatore |
| 43 | Periyakulam (Ukkadam) | Non-PA | Coimbatore |
| 44 | Perur | Non-PA | Coimbatore |
| 45 | Krishnampathy Lake | Non-PA | Coimbatore |
| 46 | Achankulam | Non-PA | Coimbatore |
| 47 | Narasampathi Lake | Non-PA | Coimbatore |
| 48 | kolarampathi | Non-PA | Coimbatore |
| 49 | Bhavani sagar Dam | Non-PA | Erode |
| 50 | Vaikkal Road Gobichettipalayam | Non-PA | Erode |
| 51 | Kalapatti | Non-PA (NLP) | Erode |
| 52 | Vellode Bird Sanctuary | PA | Erode |
| 53 | Kichagathiyur Tank Medu | Non-PA (NLP) | Coimbatore |
| 54 | Thapovanam | Non-PA (NLP) | Erode |
| 55 | RN pudur | Non-PA (NLP) | Erode |
| 56 | Thayirpalam | Non-PA | Erode |
| 57 | Ariyappampalayam | Non-PA | Erode |
| 58 | Sirumugai | Non-PA | Coimbatore |
| 59 | Bhavani Sagar | Non-PA | Erode |
| 60 | Karachi Korai | Non-PA | Erode |
| 61 | Palayam | Non-PA | Erode |
| 62 | Sathy Range office | Non-PA (NLP) | Erode |
| 63 | Ammapettai | Non-PA | Erode |
| 64 | Vardanallur | Non-PA | Erode |
| 65 | kichagathiyur 2 | Non-PA | Coimbatore |
| 66 | Ooty Lake | Non-PA (NLP) | Nilgiris |
| 67 | Koolipalayam | Non-PA | Tiruppur |
| 68 | Manikapuram | Non-PA | Tiruppur |
| 69 | Udumalaipettai | Non-PA (NLP) | Tiruppur |
| 70 | Ottukulam (Udumalpet) | Non-PA | Tiruppur |
| 71 | Periyakulam (Udumalpet) | Non-PA | Tiruppur |
| 72 | Padavalkalavai | Non-PA | Erode |
| 73 | Mettur Dam Park | Non-PA | Salem |
| 74 | Pallamalli | Non-PA | Erode |
| 75 | Ranipet Police station | Non-PA (NLP) | Vellore |
| 76 | Perunkanchi Lake | Non-PA | Vellore |
| 77 | Dhamal | Non-PA | Kancheepuram |
| 78 | Devarkulam | Non-PA | Krishnagiri |
| 79 | Kondama Lake | Non-PA | Krishnagiri |
| 80 | Vettangudi | PA | Sivaganga |
| 81 | Samanatham | Non-PA | Madurai |
| 82 | karaivetti | PA | Ariyalur |
| 83 | TVS Motors | Non-PA (NLP) | Krishnagiri |
| 84 | Thanam | Non-PA | Tiruchirapalli |
| 85 | Kottai Temple | Non-PA (NLP) | Tiruchirapalli |
| 86 | Tharanallur | Non-PA | Tiruchirapalli |
| 87 | Thiruverumbur | Non-PA | Tiruchirapalli |
| 88 | Sangaliandapuram | Non-PA | Tiruchirapalli |
| 89 | Sriramapuram | Non-PA | Tiruchirapalli |
| 90 | Puvalur | Non-PA | Tiruchirapalli |
| 91 | Nanjaisengandhi | Non-PA | Tiruchirapalli |
| 92 | Kokkuvetti | Non-PA (NLP) | Tiruchirapalli |
| 93 | Kilapudur | Non-PA (NLP) | Tiruchirapalli |
| 94 | Railway Colony | Non-PA | Tiruchirapalli |
| 95 | K.Sattanur | Non-PA | Tiruchirapalli |
| 96 | Kattuputhur | Non-PA | Tiruchirapalli |
| 97 | Varagneri, Tharanallur | Non-PA | Tiruchirapalli |
| 98 | Tamil Nadu Newsprint and Papers Limited | Non-PA (NLP) | Karur |
| 99 | Sri Meenakshi Sundareswarer Koil | Non-PA (NLP) | Karur |
| 100 | Kulithalai | Non-PA | Karur |
| 101 | Arignar Anna Zoological Park (Aviary) | PA | Kancheepuram |

Abbreviation: PA-Protected area, Non-PA- Non protected area, NLP- Non legally protected
