# Supplementary material for: Heronry distribution and site preference dynamics of tree-nesting colonial waterbirds in Tamil Nadu
Source: PeerJ. 2021 Oct 7;9:e12256. doi: 10.7717/peerj.12256 (PMC8502450; doi:10.7717/peerj.12256)
Supplement: Supplemental Information 3 [file peerj-09-12256-s003.docx]

Table S3 Colonial nesting waterbird species breeding in India (Subramanya, 1996)

| Order | Family | Common name | Scientific name | IUCN Status |
| --- | --- | --- | --- | --- |
| Pelecaniformes | Pelecanidae | Spot-billed Pelican | *Pelecanus philippensis* | NT |
|  |  | Great white Pelican | *Pelecanus onocrotalus* | LC |
|  | Ardeidae | Little Egret | *Egretta garzetta* | LC |
|  |  | Intermediate Egret | *Ardea intermedia* | LC |
|  |  | Great Egret | *Ardea alba* | LC |
|  |  | Cattle Egret | *Bubulcus ibis* | LC |
|  |  | Grey Heron | *Ardea cinerea* | LC |
|  |  | Purple Heron | *A.purpurea* | LC |
|  |  | Indian Pond-Heron | *Ardeola grayii* | LC |
|  |  | Black-crowned Night-Heron | *Nycticorax nycticorax* | LC |
|  |  | Chinese pond heron | *Ardeola bacchus* | LC |
|  |  | Western reef egret | *Egretta gularis* | LC |
|  |  | Eastern reef egret | *Egretta sacra* | LC |
|  | Threskiornithidae | Glossy Ibis | *Plegadis falcinellus* | LC |
|  |  | Black-headed Ibis | *Threskiornis melanocephalus* | NT |
|  |  | Indian Black Ibis | *Pseudibis papillosa* | LC |
|  |  | Eurasian Spoonbill | *Platalea leucorodia* | LC |
| Suliformes | Phalacrocoracidae | Little Cormorant | *Microcarbo niger* | LC |
|  |  | Indian Cormorant | *P. fuscicollis* | LC |
|  |  | Great Cormorant | *Phalacrocorax carbo* | LC |
|  | Anhingidae | Oriental Darter | *Anhinga melanogaster* | NT |
|  | Ciconiidae | Painted Stork | *Mycteria leucocephala* | NT |
|  |  | Asian Openbill | *Anastomus oscitans* | LC |
|  |  | Woolly necked stork | *Ciconia episcopus* | NT |
|  |  | Lesser adjutant stork | *Leptoptilos javanicus* | VU |
|  |  | Greater adjutant stork | *Leptoptilos dubius* | EN |

Abbreviation: NT- Near threatened, EN- Endangered, LC- Least Concern, VU- Vulnerable
