# Supplementary material for: Heronry distribution and site preference dynamics of tree-nesting colonial waterbirds in Tamil Nadu
Source: PeerJ. 2021 Oct 7;9:e12256. doi: 10.7717/peerj.12256 (PMC8502450; doi:10.7717/peerj.12256)
Supplement: Supplemental Information 4 [file peerj-09-12256-s004.docx]

**Month wise breeding details of colonial nesting waterbirds at heronries in Tamil Nadu**

| Heronry | SEP | OCT | NOV | DEC | JAN | FEB | MAR | APR | MAY |  | JUNE | JULY | AUG |
| --- | --- | --- | --- | --- | --- | --- | --- | --- | --- | --- | --- | --- | --- |
| Vedanthangal |  |  |  |  |  |  |  |  |  |  |  |  |  |
| Melmaruvathur |  |  |  |  |  |  |  |  |  |  |  |  |  |
| National Institute of Ocean Technology (NIOT) |  |  |  |  |  |  |  |  |  |  |  |  |  |
| Madras Crocodile Bank Trust |  |  |  |  |  |  |  |  |  |  |  |  |  |
| Indian Institute of Technology Madras |  |  |  |  |  |  |  |  |  |  |  |  |  |
| Otteri |  |  |  |  |  |  |  |  |  |  |  |  |  |
| Ponneri |  |  |  |  |  |  |  |  |  |  |  |  |  |
| Sri Ramachandra Medical College |  |  |  |  |  |  |  |  |  |  |  |  |  |
| Kaliveli |  |  |  |  |  |  |  |  |  |  |  |  |  |
| Kanadamangalam |  |  |  |  |  |  |  |  |  |  |  |  |  |
| Thengaithittu |  |  |  |  |  |  |  |  |  |  |  |  |  |
| Agaranallur (Banks of Kollidam River stretch) |  |  |  |  |  |  |  |  |  |  |  |  |  |
| Varagur (Banks of Kollidam River stretch) |  |  |  |  |  |  |  |  |  |  |  |  |  |
| Kodiyampalayam |  |  |  |  |  |  |  |  |  |  |  |  |  |
| Vaduvoor |  |  |  |  |  |  |  |  |  |  |  |  |  |
| Udayamarthandapuram |  |  |  |  |  |  |  |  |  |  |  |  |  |
| Tiruvarur University & surrounding |  |  |  |  |  |  |  |  |  |  |  |  |  |
| Periyakanmoi |  |  |  |  |  |  |  |  |  |  |  |  |  |
| Therthangal |  |  |  |  |  |  |  |  |  |  |  |  |  |
| Muthupet |  |  |  |  |  |  |  |  |  |  |  |  |  |
| Melselvanur-Keelaselvanur |  |  |  |  |  |  |  |  |  |  |  |  |  |
| Karankadu |  |  |  |  |  |  |  |  |  |  |  |  |  |
| Vaalai Island |  |  |  |  |  |  |  |  |  |  |  |  |  |
| Shingle Island |  |  |  |  |  |  |  |  |  |  |  |  |  |
| Sayarpuram |  |  |  |  |  |  |  |  |  |  |  |  |  |
| korampalam |  |  |  |  |  |  |  |  |  |  |  |  |  |
| Arumugamangalam |  |  |  |  |  |  |  |  |  |  |  |  |  |
| Koonthankulam |  |  |  |  |  |  |  |  |  |  |  |  |  |
| Kadankulam |  |  |  |  |  |  |  |  |  |  |  |  |  |
| Tirupadaimaruthur |  |  |  |  |  |  |  |  |  |  |  |  |  |
| Vagaikulam |  |  |  |  |  |  |  |  |  |  |  |  |  |
| Velankulam |  |  |  |  |  |  |  |  |  |  |  |  |  |
| Kariyandi |  |  |  |  |  |  |  |  |  |  |  |  |  |
| Aramaneri |  |  |  |  |  |  |  |  |  |  |  |  |  |
| Kandigaiperi |  |  |  |  |  |  |  |  |  |  |  |  |  |
| Manur |  |  |  |  |  |  |  |  |  |  |  |  |  |
| Arunthapatti |  |  |  |  |  |  |  |  |  |  |  |  |  |
| Mukkadal |  |  |  |  |  |  |  |  |  |  |  |  |  |
| Kadayanallur |  |  |  |  |  |  |  |  |  |  |  |  |  |
| Suchindram |  |  |  |  |  |  |  |  |  |  |  |  |  |
| Sulur |  |  |  |  |  |  |  |  |  |  |  |  |  |
| Vellalore |  |  |  |  |  |  |  |  |  |  |  |  |  |
| Periyakulam (Ukkadam) |  |  |  |  |  |  |  |  |  |  |  |  |  |
| Perur |  |  |  |  |  |  |  |  |  |  |  |  |  |
| Krishnampathy Lake |  |  |  |  |  |  |  |  |  |  |  |  |  |
| Achankulam |  |  |  |  |  |  |  |  |  |  |  |  |  |
| Narasampathi Lake |  |  |  |  |  |  |  |  |  |  |  |  |  |
| kolarampathi |  |  |  |  |  |  |  |  |  |  |  |  |  |
| Bhavani sagar Dam |  |  |  |  |  |  |  |  |  |  |  |  |  |
| Vaikkal Road Gobichettipalayam |  |  |  |  |  |  |  |  |  |  |  |  |  |
| Kalapatti |  |  |  |  |  |  |  |  |  |  |  |  |  |
| Vellode Bird Sanctuary |  |  |  |  |  |  |  |  |  |  |  |  |  |
| Kichagathiyur Tank Medu |  |  |  |  |  |  |  |  |  |  |  |  |  |
| Thapovanam |  |  |  |  |  |  |  |  |  |  |  |  |  |
| RN pudur |  |  |  |  |  |  |  |  |  |  |  |  |  |
| Thayirpalam |  |  |  |  |  |  |  |  |  |  |  |  |  |
| Ariyappampalayam |  |  |  |  |  |  |  |  |  |  |  |  |  |
| Sirumugai |  |  |  |  |  |  |  |  |  |  |  |  |  |
| Bhavani Sagar |  |  |  |  |  |  |  |  |  |  |  |  |  |
| Karachi Korai |  |  |  |  |  |  |  |  |  |  |  |  |  |
| Palayam |  |  |  |  |  |  |  |  |  |  |  |  |  |
| Sathy Range office |  |  |  |  |  |  |  |  |  |  |  |  |  |
| Ammapettai |  |  |  |  |  |  |  |  |  |  |  |  |  |
| Vardanallur |  |  |  |  |  |  |  |  |  |  |  |  |  |
| kichagathiyur 2 |  |  |  |  |  |  |  |  |  |  |  |  |  |
| Ooty Lake |  |  |  |  |  |  |  |  |  |  |  |  |  |
| Koolipalayam |  |  |  |  |  |  |  |  |  |  |  |  |  |
| Manikapuram |  |  |  |  |  |  |  |  |  |  |  |  |  |
| Udumalaipettai |  |  |  |  |  |  |  |  |  |  |  |  |  |
| Ottukulam (Udumalpet) |  |  |  |  |  |  |  |  |  |  |  |  |  |
| Periyakulam (Udumalpet) |  |  |  |  |  |  |  |  |  |  |  |  |  |
| Padavalkalavai |  |  |  |  |  |  |  |  |  |  |  |  |  |
| Mettur Dam Park |  |  |  |  |  |  |  |  |  |  |  |  |  |
| Pallamalli |  |  |  |  |  |  |  |  |  |  |  |  |  |
| Ranipet Police station |  |  |  |  |  |  |  |  |  |  |  |  |  |
| Perunkanchi Lake |  |  |  |  |  |  |  |  |  |  |  |  |  |
| Dhamal |  |  |  |  |  |  |  |  |  |  |  |  |  |
| Devarkulam |  |  |  |  |  |  |  |  |  |  |  |  |  |
| Kondama Lake |  |  |  |  |  |  |  |  |  |  |  |  |  |
| Vettangudi |  |  |  |  |  |  |  |  |  |  |  |  |  |
| Samanatham |  |  |  |  |  |  |  |  |  |  |  |  |  |
| karaivetti |  |  |  |  |  |  |  |  |  |  |  |  |  |
| TVS Motors |  |  |  |  |  |  |  |  |  |  |  |  |  |
| Thanam |  |  |  |  |  |  |  |  |  |  |  |  |  |
| Kottai Temple |  |  |  |  |  |  |  |  |  |  |  |  |  |
| Tharanallur |  |  |  |  |  |  |  |  |  |  |  |  |  |
| Thiruverumbur |  |  |  |  |  |  |  |  |  |  |  |  |  |
| Sangaliandapuram |  |  |  |  |  |  |  |  |  |  |  |  |  |
| Sriramapuram |  |  |  |  |  |  |  |  |  |  |  |  |  |
| Puvalur |  |  |  |  |  |  |  |  |  |  |  |  |  |
| Nanjaisengandhi |  |  |  |  |  |  |  |  |  |  |  |  |  |
| Kokkuvetti |  |  |  |  |  |  |  |  |  |  |  |  |  |
| Kilapudur |  |  |  |  |  |  |  |  |  |  |  |  |  |
| Railway Colony |  |  |  |  |  |  |  |  |  |  |  |  |  |
| K.Sattanur |  |  |  |  |  |  |  |  |  |  |  |  |  |
| Kattuputhur |  |  |  |  |  |  |  |  |  |  |  |  |  |
| Varagneri, Tharanallur |  |  |  |  |  |  |  |  |  |  |  |  |  |
| Tamil Nadu Newsprint and Papers Limited |  |  |  |  |  |  |  |  |  |  |  |  |  |
| Sri Meenakshi Sundareswarer Koil |  |  |  |  |  |  |  |  |  |  |  |  |  |
| Kulithalai |  |  |  |  |  |  |  |  |  |  |  |  |  |
| Arignar Anna Zoological Park (Aviary) |  |  |  |  |  |  |  |  |  |  |  |  |  |
