# Supplementary material for: Heronry distribution and site preference dynamics of tree-nesting colonial waterbirds in Tamil Nadu
Source: PeerJ. 2021 Oct 7;9:e12256. doi: 10.7717/peerj.12256 (PMC8502450; doi:10.7717/peerj.12256)
Supplement: Supplemental Information 5 [file peerj-09-12256-s005.docx]

Table S5 Percentage of occurrence

| Species | No of sites nesting recorded (101) | % of sites |
| --- | --- | --- |
| Little Cormorant | 80 | 79.21 |
| Little Egret | 67 | 66.34 |
| Black-crowned Night-Heron | 52 | 51.49 |
| Indian Pond-Heron | 44 | 43.56 |
| Intermediate Egret | 30 | 29.70 |
| Oriental Darter | 29 | 28.71 |
| Grey Heron | 26 | 25.74 |
| Indian Cormorant | 24 | 23.76 |
| Black-headed Ibis | 19 | 18.81 |
| Asian openbill | 17 | 16.83 |
| Spot-billed Pelican | 16 | 15.84 |
| Eurasian Spoonbill | 14 | 13.86 |
| Painted Stork | 14 | 13.86 |
| Cattle Egret | 11 | 10.89 |
| Purple Heron | 11 | 10.89 |
| Glossy Ibis | 11 | 10.89 |
| Large Egret | 10 | 9.90 |
| Great Cormorant | 5 | 4.95 |
| Indian Black Ibis | 2 | 1.98 |
