# Supplementary material for: Heronry distribution and site preference dynamics of tree-nesting colonial waterbirds in Tamil Nadu
Source: PeerJ. 2021 Oct 7;9:e12256. doi: 10.7717/peerj.12256 (PMC8502450; doi:10.7717/peerj.12256)
Supplement: Supplemental Information 6 [file peerj-09-12256-s006.docx]

**Table S6 Heronries with breeding colonial nesting waterbird species**

| **S.No** | **Name of the Heronry** | **No of Species (2017-2019)** | **Breeding species** |
| --- | --- | --- | --- |
| 1 | Vedanthangal | 16 | OBS,SPB,LC,IC,LE,IE,LargeE,CE,NH,PH, GH,GlosIB, PS, Pelican,BHI,Darter |
| 2 | Melmaruvathur | 12 | SPB,LC,IC,LE,IE,NH,PH,GH,PS, Pelican,BHI,Darter |
| 3 | NIOT | 9 | LC, NH,PH, GH,GlosIB,PS,Pelican,BHI,Darter |
| 4 | Madras crocodile bank trust | 3 | LC,LE, NH |
| 5 | IITM | 2 | LC, Darter |
| 6 | Otteri | 12 | SPB,LC,IC,LE,IE,CE,NH,PH, GH, PS,BHI,Darter |
| 7 | Ponneri | 10 | OBS,LC,LE,IE,CE, PurH, NH,PH, GlosIB, BHI |
| 8 | SRMC | 4 | LC,LE,IE, NH. |
| 9 | Kaliveli | 11 | SPB,LC,IC,LE,IE,NH,GH, PS, Pelican,BHI,Darter |
| 10 | Kandamangalam | 1 | PH |
| 11 | Thengaithittu | 5 | LC,LE,IE,NH,PH |
| 12 | Agaranallur | 1 | PH |
| 13 | Varagur | 1 | PH |
| 14 | Kodiyampalayam | 7 | LC, LE,IE, LargeE, NH, PH, GH |
| 15 | Vaduvoor | 14 | OBS,LC,IC,GC,LE,IE,LargeE,PurH,NH,PH, GH,GlosIB,BHI,Darter |
| 16 | Udayamarthandapuram | 14 | OBS,LC,IC,LE,IE,LargeE,CE,PurH,NH,PH, GH,GlosIB,BHI,Darter |
| 17 | Tiruvarur University & surrounding | 2 | LC, LE |
| 18 | Periyakanmoi | 8 | OBS,LC,LE,NH,PH,GH,BHI,Darter |
| 19 | Therthangal | 2 | SPB,PS |
| 20 | Muthupet | 4 | PS, Darter, LE, NH |
| 21 | Melselvanur-Keelaselvanur | 1 | Pelican |
| 22 | Karankadu | 4 | LC,LE,IE,LargeE |
| 23 | Vaalai island | 2 | LE,GH |
| 24 | Shingle island | 2 | LE,GH |
| 25 | Sayarpuram | 1 | Pelican |
| 26 | korampalam | 1 | Pelican |
| 27 | Arumugamangalam | 7 | LC,LE,IE,CE, PurH, NH,PH |
| 28 | Koonthakulam | 16 | OBS,SPB,LC,IC,LE,IE,LargeE,CE,NH,PH, GH,GlosIB,PS,Pelican,BHI,Darter |
| 29 | Kadankulam | 3 | SPB,Pelican,PS |
| 30 | Tirupadaimaruthur | 7 | LC,LE,LargeE,PH, GH, PS, Pelican |
| 31 | Vagaikulam | 15 | OBS,LC,IC,LE,IE,LargeE,CE,PurH,NH,PH, GH,GlosIB, Pelican,BHI, Darter |
| 32 | Velankulam | 9 | OBS,LC,LE,IE,PurHeron,NH,PH,GlosIB,Darter |
| 33 | Kariyandi | 3 | LC, Black Ibis, Darter |
| 34 | Aramaneri | 1 | Black Ibis |
| 35 | Kandigaiperi | 1 | SPB |
| 36 | Manur | 10 | OBS,SPB,LC,IC,LE,CE,PH,GH,BHI,Darter |
| 37 | Arunthapatti | 9 | OBS,SPB,LC,LE,IE,NH,GH,BHI,Darter |
| 38 | Mukkadal | 2 | LC,LE |
| 39 | Kadyanallur | 2 | LC, IC |
| 40 | Suchindram | 5 | OBS,LC,Pelican,BHI,Darter |
| 41 | Sulur | 7 | LC,IC,PurH,NH,PH,GH,Darter |
| 42 | Vellalore | 6 | SPB,LC,IC,PH,PS,Pelican |
| 43 | Periyakulam (ukkadam) | 4 | LC,NH,PH,GH |
| 44 | Perur | 6 | LC,LE,IE,PurH,NH,Darter |
| 45 | Krishnampathy lake | 3 | LC, IC, LE |
| 46 | Achankulam | 11 | OBS,LC,IC,LE,IE,PurH,NH,PH, GH,Pelican,Darter |
| 47 | Narasampathi Lake | 4 | LC,IC,NH,Darter |
| 48 | kolarampathi | 5 | LC,LE,IE,PH,Darter |
| 49 | Bhavani sagar Dam | 3 | LC,LE,GH |
| 50 | Vaikkal Road GOBI | 3 | LC,LE,NH |
| 51 | Kalapatti | 3 | LC,LE,PH |
| 52 | Vellode BS | 13 | SPB,LC,IC,GC,LE,IE,LargeE,CE,NH,PH, GH,BHI,Darter |
| 53 | Kichagathiyur Tank medu | 4 | LC,LE,IE,NH |
| 54 | Thapovanam | 2 | LC,LE |
| 55 | R N pudur | 2 | LE,NH |
| 56 | Thayirpalam | 1 | LC |
| 57 | Ariyappampalayam | 3 | LC,NH,PH |
| 58 | Sirumugai | 1 | LC |
| 59 | Bhavani sagar | 4 | LC,LE,IE,NH |
| 60 | Karachi korai | 4 | LC,LE,NH,PH |
| 61 | Palayam | 2 | LC,NH |
| 62 | Sathy Range office | 4 | LC,IC,IE,NH |
| 63 | Ammapettai | 2 | IC,NH |
| 64 | Vardanallur | 4 | LC,LE,NH,PH |
| 65 | kichagathiyur 2 | 1 | LC |
| 66 | Ooty Lake | 1 | GC |
| 67 | Koolipalayam | 7 | LC, LE, PurH, NH, PH, GH, Darter |
| 68 | Manikapuram | 4 | LC,IC,LE,PH |
| 69 | Udumalaipettai | 3 | LC,IC,LE |
| 70 | Ottukulam | 4 | LC,LE,NH,PH |
| 71 | Periyakulam | 3 | LC,LE,PH |
| 72 | Padavalkalavai | 1 | NH |
| 73 | Mettur dam park | 5 | LC,LE,IE,PH,NH |
| 74 | Pallamalli | 1 | LE |
| 75 | Ranipet Police station | 4 | LC,LE,NH,GH |
| 76 | Perunkanchi | 3 | LC,LE,NH |
| 77 | Dhamal | 6 | LC,LE,NH,CE,PH,GlosIB |
| 78 | Devarkulam | 3 | PS,BHI,PH |
| 79 | Kondama lake | 1 | GC |
| 80 | Vettangudi BS | 8 | OBS,LC,LE,IE,NH,PH,BHI,Darter |
| 81 | Samanatham | 11 | OBS,SPB,LC,IC,LE,NH,PH,GH,GlosIB,BHI, Darter |
| 82 | karaivetti BS | 14 | OBS,SPB,LC,IC,GC,LE,IE,PurH,NH,PH, GH,Pelican,BHI,Darter |
| 83 | TVS motors | 6 | OBS,LC,LargeE,NH,GH,PS |
| 84 | Devathanam | 2 | LC,LE |
| 85 | Kottai temple | 2 | LC,LE |
| 86 | Tharanallur | 3 | LC,IC,LE |
| 87 | Thiruverumbur | 6 | LC,IC,LE,IE,NH,Darter |
| 88 | Sangaliandapuram | 3 | LC,LE,NH |
| 89 | Sriramapuram | 3 | LC,LE,NH |
| 90 | Puvalur | 3 | LC,CE,Darter |
| 91 | Nanjaisengandhi | 6 | OBS,LC,LE,IE,NH,GlosIB |
| 92 | Kokku vetti | 3 | LC,LE,IE |
| 93 | Kilapudur | 2 | LC,LE |
| 94 | Rly colony | 3 | LC,LE,PH |
| 95 | K. Sattanur | 2 | LC,LE |
| 96 | Kattuputhur | 3 | LC,LE,PH |
| 97 | Varagneri, Tharanallur | 3 | LC,LE,PH |
| 98 | TNPL | 3 | LC,LE,NH |
| 99 | SriMeenakshi Sunderaswarer Koil | 4 | LC,LE,IE,PH |
| 100 | Kulithalai | 1 | LC |
| 101 | Aviary (AAZP) | 4 | PS,pelican,PH,NH |

Abbrevations: OBS- Asian openbill, PS- Painted Stork, SPB-Eurasian Spoonbill, Pelican- Spotbilled Pelican, LE-Little Egret, IE- Intermediate Egret, LargeE- Large Egret,CE- Cattle Egret, LC- Little Cormorant, IC- Indian Cormorant, GC- Great Cormorant, BHI- Black headed Ibis, Black Ibis- Indian Black Ibis, GlosIB- Glossy Ibis, PurH- Purple Heron, PH- Pond Heron, NH- Black crowned Night Heron, GH- Grey Heron, Darter- Oriental Darter
