# Supplementary material for: Heronry distribution and site preference dynamics of tree-nesting colonial waterbirds in Tamil Nadu
Source: PeerJ. 2021 Oct 7;9:e12256. doi: 10.7717/peerj.12256 (PMC8502450; doi:10.7717/peerj.12256)
Supplement: Supplemental Information 7 [file peerj-09-12256-s007.docx]

Table S7: Details regarding the heronries along the roadsides

| 1. Mukkadal |
| --- |
| 1. Kalapatti |
| 1. Thayirpallam |
| 1. Ariyappampalayam |
| 1. Sirumugai |
| 1. Karachikorai |
| 1. Palayam |
| 1. Ammapettai |
| 1. Varthanallur |
| 1. Padavalkalavai |
| 1. Pallamalli |
| 1. Kattuputhur |
