# Supplementary figures and images for: Heronry distribution and site preference dynamics of tree-nesting colonial waterbirds in Tamil Nadu
Source: PeerJ. 2021 Oct 7;9:e12256. doi: 10.7717/peerj.12256 (PMC8502450; doi:10.7717/peerj.12256)

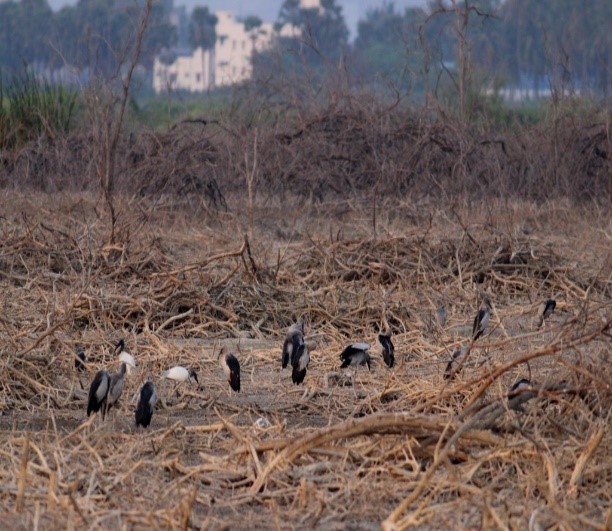

Supplement: Supplemental Information 9 [file peerj-09-12256-s009.jpg]

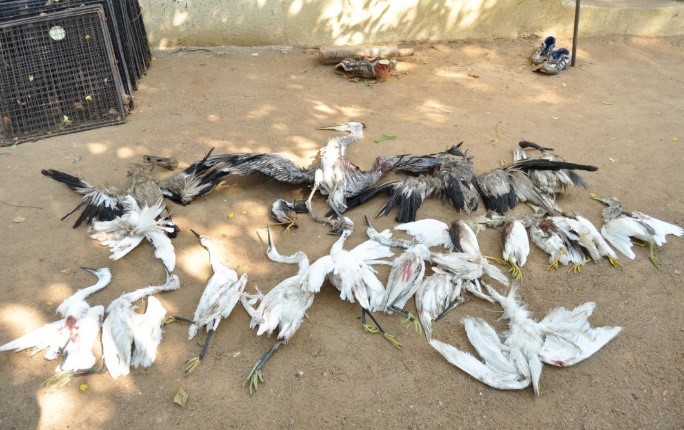

Supplement: Supplemental Information 10 [file peerj-09-12256-s010.jpg]

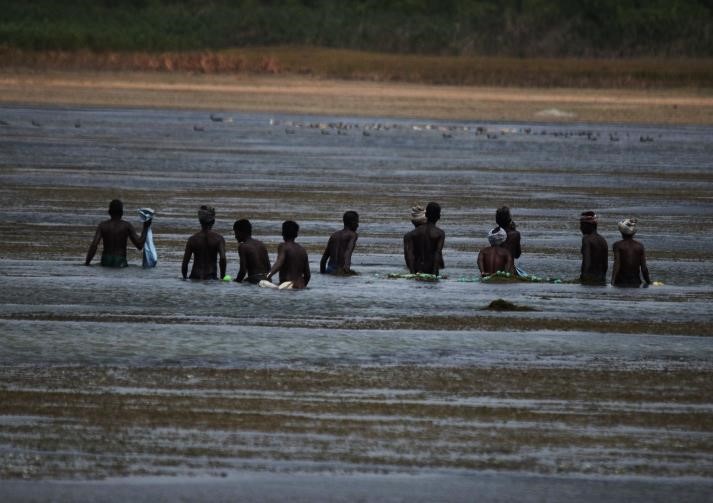

Supplement: Supplemental Information 11 [file peerj-09-12256-s011.jpg]

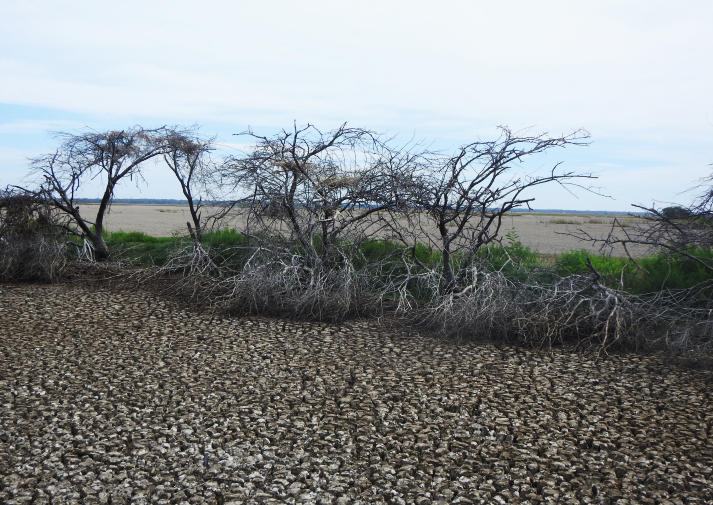

Supplement: Supplemental Information 12 [file peerj-09-12256-s012.png]
