# Supplementary material for: Heronry distribution and site preference dynamics of tree-nesting colonial waterbirds in Tamil Nadu
Source: PeerJ. 2021 Oct 7;9:e12256. doi: 10.7717/peerj.12256 (PMC8502450; doi:10.7717/peerj.12256)
Supplement: Supplemental Information 13 — Abstract of the article in Tamil (unreviewed). [file peerj-09-12256-s013.pdf]

மரங்களில் பெரும் குழுக்களாக கூடு கட்டி வாழும் நீர் பறவைகளின் பரவல் மற்றும் அவற்றின் வாழ்விடத்தேர்வு குறித்து தமிழ்நாட்டில் மேற்கொள்ளப்பட்ட கள ஆய்வு

ச.ஜெ. த. பிராங்க், கோ. வீ. கோபி மற்றும் பி. பாண்டவ்
இந்திய காணுயிர் நிறுவனம், டெராடூன், உத்திரகாண்ட் மாநிலம் -248 001

தொடர்புக்கு: gopigv@wii.gov.in

சுருக்கம் (Abstract):

ஆசியா போன்ற மக்கட்தொகை கூடுதலாக உள்ள கண்டங்களில், புவிநிலை மாற்றமும் மனித இடையூறுகளும் நீர் நிலைகளின் பரவலை வெகுவாகத் தீர்மானிக்கிறது. இந்தியாவின் பல்வேறு உயிர் புவியியல் மண்டலங்களில் (Biogeographic Zones) பல்வேறு வகையான நீர் நிலைகள் அமைந்துள்ளன. இந்நீர்நிலைகள் மனித சமுதாய மற்றும் பல்வகையான காணுயிர்களின் உயிர்சூழல் ஆதாரமாக விளங்குகிறது. இருப்பினும் நீர் நிலைகள் பல்வேறு வகையான ஆச்சுறுதல்களால் பாதிக்கப்பட்டு அற்றுப்போகும் நிலையில் உள்ளன. மனித இடையூறுகள் சார்ந்த ஆச்சுறுதல்களால், முக்கியமாக மரங்களில் பெரும் குழுக்களாக கூடுகட்டி இனபெருக்கம் செய்யும் நீர் சார்ந்த புள்ளினங்கள் (tree-nesting colonial waterbirds) பெரும் பாதிப்படைய வாய்ப்புகள் உள்ளது. இவ்வகை பெரும் குழுக்களாக கூடுகட்டி வாழும் புகலிடங்கள், ஹெரான்ரி (Heronry) என்று ஆங்கிலத்தில் அழைக்கப்படுகிறது. ஹெரான்ரி உள்ள பகுதிகளுக்கு ஏற்படும் ஆச்சுறுதல்களால் அவை சார்ந்த புள்ளினங்கள் மொத்தமாக வேறு இடங்களுக்கு இடம் பெயரும் அபாயம் உள்ளது. ஹெரான்ரி பாதுகாப்பு, புள்ளினம் மற்றும் அவை சார்ந்த வாழ்விடங்களின் பேணலின் ஒரு முக்கிய அம்சமாகும். ஹெரான்ரி பாதுகாப்பின் மூலம் பல்லுயிர் பேணல் (Biodiversity Conservation), குடிகளுக்கு தேவையான நீர்நிலை பாதுகாப்பு, மற்றும் சுற்றுச்சூழல் அமைப்பு சேவைகள் (Ecosystem Services) கிடைப்பதால், அரசு ஹெரான்ரி பாதுகாப்பிற்கு முனைப்புடன் பல்வேறு திட்டங்களை முன்னிறுத்தி வருகின்றது. நீர்நிலை மேலாண்மை மற்றும் பாதுகாப்பில் தமிழ்நாடு தொன்றுதொட்டு முன்னோடியாக இருந்துவருவதால் ஹெரான்ரிகளின் தற்போதைய பரவலை அறிவியல் ரீதியாக அறிந்து கொள்ளும் நோக்கொடு இந்திய காணுயிர் நிறுவனம் (Wildlife Institute of India) தமிழ்நாடு வனத்துறையின் உதவியோடு 2017-2019ல் கள ஆய்வுகளை மாநிலம் முழுவதும் மேற்கொண்டது.

இந்த ஆய்வில் திரட்டப்பட்ட தரவுகள் நவீன கணித மற்றும் புள்ளியல் மாதிரிகள் (mathematical and statistical models) கொண்டு ஆராயப்பட்டன. இந்த முடிவுகளின்படி, தமிழ் நாட்டில் அப்போதிருந்த 32 மாவட்டங்களில், 22ல் 101 ஹெரான்ரிக்கள் இருப்பது தெரிய வந்துள்ளது. அவற்றில் 19% ஹெரான்ரிக்கள் பாதுகாக்கப்பட்ட பகுதிகளாகவும், எஞ்சிய 81% ஹெரான்ரிக்கள் அப்பகுதிகளுக்கு வெளியேவும் இருப்பது கண்டறியப்பட்டுள்ளது. சுமார் 50 விழுக்காடு ஹெரான்ரிக்கள் நகர்புரங்களிலும் மக்கள் பயன்பாட்டில் உள்ள பகுதிகளிலும் இருக்கின்றன. தமிழக ஹெரான்ரிக்களில் சிறிய நீர்க்காகம் (லிட்டில் கார்மோரண்ட் *Microcarbo niger*) மிகவும் பரவலான புள்ளினமாக இருப்பது தெரிய வந்துள்ளது. கூடு கட்ட, பறவைகள் 23 வகையான மரங்களை தேர்வு செய்கின்றன. இதில் முதன்மையாக பறவைகள் பயன்படுத்துவது கருவேல மரங்களாகும் (*Vachellia nilotica*). மேலும் புள்ளினங்கள், பெரும் நீர் நிலைகளும், ஆறுகளும் அவை சார்ந்த இதர வாழிட பகுதிகளை அதிக அளவில் பயன்படுத்துவது தெரிய வந்துள்ளது. இதற்கு முன்பு 2005ல் மேற்கொள்ளப்பட்ட கள ஆய்வோடு ஒப்பிட்டு பார்க்கும் பொழுது, இடைப்பட்ட காலத்தில் பல ஹெரான்ரிக்கள் அற்று போனது தெரிய வந்துள்ளது.

நீர் சார்ந்த புள்ளின திரள்களை (Waterbird populations), அறிவியல் சார்ந்த குறியீடுகள் (Indices) மூலமாக தொடர்ந்து கண்காணிப்பது இன்றியமையதாது என்பதை இவ்வாய்வு சுட்டிக்காட்டுகிறது. மேலும், நீர்நிலைகளை சார்ந்து இருக்கும் பறவைகள் பேணலுக்கு, நீர்நிலை ஆக்கிரமைப்பை கண்காணித்தல், கள்ள வேட்டையிலிருந்து பாதுகாத்தல், மற்றும் கூடு கட்ட பறவைகள் பயன்படுத்தும் மரங்களை பாதுகாத்தல் மிக அவசியம் என்பதை இவ்வாய்வு உணர்த்துகிறது. பெரும்பாலான ஹெரான்ரிக்கள் நகர்புரங்களிலும், மக்கள் பயன்பாட்டில் உள்ள பகுதிகளிலும் இருப்பதால் அவற்றின் பேணலில் மக்களின் பங்களிப்பு ஹெரான்ரி பாதுகாப்பிற்கு இன்றியமையாதது.

இது போன்ற கள ஆய்வு நாடு முழுவதும் மேற்கொள்ளப்பட்டால் நாடு தழுவிய நீர்நிலை மேலாண்மை மற்றும் அவை சார்ந்த பல்லுயிர் பேணலுக்கு வழி வகுக்கும்.

முக்கிய வார்த்தைகள் (Keywords): பருவமழை, பாதுகாக்கப்பட்ட பகுதிகள், நீர்நிலைகள், மனித இடையூறுகள், மரங்களில் பெரும் குழுக்களாக கூடு கட்டி வாழும் நீர் பறவைகள்
